# Self-supervised learning for inflammatory response pattern recognition in physical education and exercise interventions

**DOI:** 10.3389/fphys.2026.1891692

**Published:** 2026-10-28

**Authors:** Shilian Xie

**Affiliations:** Department of Public Basic Courses, Department of Physical Education,Shanghai University of Finance and Economics Zhejiang College, Jinhua Zhejiang, China

**Keywords:** inflammatory response, pattern recognition, physical education, PhysicalForwardPersistent model, self-supervised learning

## Abstract

This study proposes PhysicalForwardPersistent, a self-supervised learning framework for recognizing inflammatory-response patterns associated with physical education and exercise interventions. The method operates on structured multivariate physiological sequences containing inflammation-related biomarkers, physiological measurements, and exercise descriptors, separating label-free representation learning from labeled downstream evaluation. PhysicalForwardPersistent integrates three components: a Physical Consistency Encoder that learns stable representations under physiologically admissible perturbations, a Forward Consistency Projector that models temporal latent-state evolution, and a Persistent Relation Extractor that preserves stable relationships among physiological profiles. The framework was evaluated on four datasets: the Physical Activity Inflammation Response Dataset, Exercise Induced Cytokine Patterns Dataset, Self-Supervised Learning Inflammation Dataset, and Physical Education Intervention Biomarker Dataset. These datasets include approximately 1,000 participants and 8,000 physiological samples, with 14–24 input variables and 2–5 temporal observations per sequence. Using participant-level separation and repeated runs, PhysicalForwardPersistent achieved accuracies of 89.23%-90.23% and macro-F1 scores of 88.34%-89.34%. Compared with the strongest baseline, it improved Accuracy by up to 1.78 percentage points and macro-F1 by up to 1.89 percentage points, with significant improvements after multiple-comparison correction. Ablation experiments confirmed that all three components contributed positively. These results demonstrate the value of jointly modeling representation stability, temporal dynamics, and relational structure for inflammatory-response pattern recognition, while further biological and prospective validation is required before practical deployment.

## 1 Introduction

Physical education and structured exercise influence not only physical fitness but also a broad range of immune and inflammatory processes. Exercise-associated inflammation should not be regarded as a uniformly adverse response: a single exercise bout can induce transient changes in circulating cytokines and immune-cell activity, whereas repeated training may produce different or more heterogeneous adaptations in basal inflammatory status. The magnitude and direction of these responses depend on factors such as exercise intensity, exercise modality, participant characteristics, and the timing of measurements relative to exercise and recovery Islam et al. (2024); Cho et al. (2022); Vázquez-Lorente et al. (2025). Understanding these dynamic responses is important for designing physical education interventions that balance training stimulus, recovery, and physiological safety.

From a physiological perspective, exercise-induced inflammation involves coordinated interactions among contracting skeletal muscle, circulating immune cells, metabolic stress, and systemic inflammatory signaling. Interleukin-6 (IL-6) is one of the most responsive exercise-associated cytokines and can increase rapidly following physical exertion, with its magnitude influenced by exercise intensity and pattern Islam et al. (2024); Cho et al. (2022). Tumor necrosis factor-alpha (TNF-*α*) and interleukin-1 beta (IL-1*β*) provide additional information about pro-inflammatory activity, although their responses can vary substantially according to the exercise protocol and physiological condition Cho et al. (2022). In parallel, anti-inflammatory mediators such as IL-10 contribute to counter-regulatory immune signaling, and circulating C-reactive protein (CRP) is frequently assessed together with cytokines to characterize systemic inflammatory status Islam et al. (2024). Consequently, inflammatory response patterns are more appropriately interpreted as multivariate trajectories involving baseline status, exercise-induced perturbation, and subsequent recovery rather than as changes in a single biomarker at an isolated time point Islam et al. (2024).

This temporal perspective is also important for distinguishing acute exercise responses from chronic inflammatory adaptation. Acute exercise can produce rapid and intensity-dependent changes in cytokine concentrations and cellular immune signaling, and these changes may partly resolve or change direction during the post-exercise recovery period Islam et al. (2024); Cho et al. (2022). In contrast, longer-term exercise training does not necessarily produce uniform reductions in every circulating inflammatory biomarker, emphasizing that chronic inflammatory status depends on training dose, population characteristics, baseline physiology, and the biomarker under consideration Vázquez-Lorente et al. (2025). Therefore, identifying physiologically meaningful inflammatory patterns requires simultaneous consideration of biomarker composition, exercise characteristics, and temporal evolution. Analyzing such data computationally is challenging because physiological datasets commonly contain multiple biomarkers and vital-sign measurements collected longitudinally, while reliable outcome annotations may be substantially more limited than the amount of available physiological data. Recent studies on cardiac signals and structured clinical time series have demonstrated that self-supervised learning can exploit unlabeled physiological observations to learn representations that remain useful for downstream prediction tasks Kiyasseh et al. (2021); Raghu et al. (2023). Temporal structure itself can also provide a self-supervisory signal, because the ordering and progression of longitudinal observations contain information that may be relevant to subsequent physiological states Agrawal et al. (2022). These findings make self-supervised representation learning particularly attractive for exercise-associated inflammatory data, where repeated physiological measurements can be available even when class annotations are comparatively expensive or task-specific. Nevertheless, directly transferring existing self-supervised approaches to inflammatoryresponse recognition remains nontrivial. Exercise-associated physiological data require a model to preserve information that is stable under measurement variation while simultaneously representing changes occurring across exercise and recovery stages. Moreover, relationships among physiological response profiles may contain information that is not captured by treating individual observations independently. Existing self-supervised studies in physiological and clinical domains establish the feasibility of contrastive and temporally structured representation learning, but they do not specifically address the joint modeling of physical consistency, forward physiological evolution, and persistent inter-sample relationships for inflammatory-response recognition in physical education interventions Kiyasseh et al. (2021); Agrawal et al. (2022); Raghu et al. (2023).

To address these challenges, we propose *PhysicalForwardPersistent*, a self-supervised framework for inflammatory-response pattern recognition using structured multivariate physiological sequences collected in the context of physical education and exercise interventions. The framework consists of three complementary components: Physical Consistency Encoding, which promotes stable representations under physiologically admissible perturbations; Forward Consistency Projection, which exploits the temporal ordering of physiological observations to model subsequent latent states; and Persistent Relation Extraction, which preserves stable relationships among physiological response profiles. During selfsupervised representation learning, inflammatory-response class labels are not used. Ground-truth labels are introduced only during downstream classification and evaluation, thereby maintaining a clear distinction between label-free physiological representation learning and labeled inflammatory-response recognition. We summarize the main contributions of this study as follows:

- We develop a self-supervised framework specifically structured around multivariate physiological observations associated with physical education interventions. The framework integrates physical consistency, forward temporal consistency, and persistent relational preservation to capture complementary characteristics of exercise-associated inflammatory responses.
- We formulate temporal inflammatory-response modeling as latent physiological state evolution, allowing the chronological structure of baseline, exercise, post-exercise, and recovery observations to provide self-supervisory information without requiring inflammatory class labels during representation learning.
- We evaluate PhysicalForwardPersistent across four physiological datasets under a unified participantlevel experimental protocol. Comparative experiments, repeated-run statistical analysis, and controlled ablation studies are used to assess both overall predictive performance and the individual contribution of the three self-supervised learning mechanisms.

## 2 Related work

### 2.1 Self-supervised learning in education

Self-supervised learning has gained significant attention in recent years as a method for leveraging unlabeled data to train machine learning models, particularly in domains where labeled datasets are scarce or costly to obtain. In the context of physical education, this approach enables the development of systems capable of identifying patterns in students’ physical activities and responses, thereby enhancing teaching interventions Liu et al. (2022a). By learning from the intrinsic structure of data, self-supervised models can adapt to dynamic environments, making them suitable for applications requiring continuous monitoring and adjustment Wang et al. (2021). These models excel in processing high-dimensional data, such as video recordings or sensor outputs from wearable devices, which are commonly used in physical education Mokmin and Rassy (2024). For example, they can analyze biomechanical movements during exercises to identify areas for improvement, allowing educators to tailor their teaching strategies to individual needs Xu and Sun (2023). Furthermore, self-supervised learning facilitates the creation of personalized learning experiences by adapting to the unique pace and style of each student, which is particularly important in physical education where physical abilities and motivations vary widely Lubis and Handayani (2022). Real-time feedback generated by these systems can help students refine their performance and achieve fitness goals more efficiently Wu et al. (2021). Beyond practical applications, self-supervised learning also supports research into physiological responses to physical activity, such as inflammation, enabling the design of interventions that optimize recovery and overall health outcomes Aldenaini et al. (2020). By uncovering patterns in these responses, educators can develop programs that minimize adverse effects and enhance the benefits of physical education Fletcher et al. (2021).

### 2.2 Inflammatory response in physical activity

The inflammatory response is a fundamental physiological process triggered by physical activity, playing a crucial role in recovery and adaptation Whitehead (2020). Investigating the patterns of inflammation in individuals engaged in physical education provides valuable insights for optimizing training regimens and reducing potential risks Merma-Molina et al. (2023). Physical activity induces a spectrum of inflammatory responses mediated by biomarkers such as cytokines and acute-phase proteins, which vary based on factors like age, fitness level, and genetic predisposition Singh et al. (2020). By analyzing these biomarkers, researchers can identify correlations between specific types of physical activity and the intensity of inflammation, offering guidance for designing effective interventions dos Santos Cardoso et al. (2022). Recognizing these patterns enables the development of personalized training programs that account for individual variability, ensuring that participants achieve fitness gains while minimizing inflammation-related risks Sharma et al. (2024). For individuals with heightened inflammatory responses, tailored regimens can mitigate excessive inflammation and promote safer engagement in physical activities Czajkowska et al. (2021). Understanding inflammation patterns plays a crucial role in developing strategies to enhance recovery, given that excessive or prolonged inflammation may impede healing and result in chronic conditions Neshat et al. (2024). By identifying optimal recovery patterns, educators can design interventions that support efficient adaptation and long-term health Wang et al. (2025). The implications of this research extend beyond physical education, as chronic inflammation is linked to various health issues, including cardiovascular disease and metabolic disorders Majrashi et al. (2024).

### 2.3 Pattern recognition in physical education

Pattern recognition is a cornerstone of machine learning, enabling the identification of regularities and structures within complex datasets Medzhitov (2021). In physical education, this capability is applied to analyze diverse data types, such as motion capture, video recordings, and physiological measurements, to improve teaching interventions and student outcomes Hannoodee and Nasuruddin (2024). Movement analysis is a key application, where pattern recognition algorithms process data from wearable sensors or motion capture systems to detect deviations from optimal techniques, allowing educators to provide precise feedback. This is particularly critical in activities requiring proper form to prevent injuries and enhance performance Liu et al. (2022a). Physiological monitoring is another area where pattern recognition proves valuable, as it can analyze metrics like heart rate, respiration, and muscle activity to identify signs of fatigue or overtraining Wang et al. (2021). Such insights enable adjustments to training regimens, ensuring safe and effective physical activity Mokmin and Rassy (2024). Adaptive learning systems in physical education also benefit from pattern recognition, as algorithms can continuously analyze student data to identify learning preferences and tailor teaching methods accordingly Xu and Sun (2023). This customization addresses the diverse needs and abilities of students, enhancing the overall effectiveness of educational programs Lubis and Handayani (2022). Furthermore, pattern recognition supports research into the interplay between physical activity and health outcomes, such as inflammation and recovery, by uncovering relationships that inform evidence-based practices Wu et al. (2021). These findings contribute to the design of interventions that optimize physical performance while promoting long-term health and well-being Aldenaini et al. (2020). The integration of pattern recognition into physical education represents a significant advancement, offering tools to refine teaching strategies, improve student engagement, and support innovative research Fletcher et al. (2021).

## 3 Method

### 3.1 Overview

This section presents an overview of the proposed PhysicalForwardPersistent framework for selfsupervised representation learning and inflammatory-response pattern recognition in physical education interventions. The framework is designed for structured multivariate physiological observations containing inflammation-related biomarkers, physiological measurements, and exercise-related descriptors collected across one or more stages of an exercise or physical education intervention. Rather than directly learning a supervised mapping from physiological inputs to inflammatory class labels, the proposed approach first exploits the intrinsic structure and temporal organization of the physiological data to learn informative latent representations without using inflammatory-response labels.

Section 3.2 formalizes the learning problem and introduces the notation used throughout the proposed framework. For the *i*-th physiological sample, the input is represented as a multivariate sequence


$${\text{X}}_{i}=[{\text{x}}_{i,1},\ldots ,{\text{x}}_{i,T_{i}}]\in {\mathbb{R}}^{T_{i}\times d},$$

where *T_i_*denotes the number of available temporal observations and *d* denotes the number of input variables after preprocessing. The proposed framework learns a latent sequence


$${\text{Z}}_{i}=[{\text{z}}_{i,1},\ldots ,{\text{z}}_{i,T_{i}}],\;\;{\text{z}}_{i,t}\in {\mathbb{R}}^{k},$$

such that the resulting representation captures information that is stable under physiologically admissible perturbations, predictive of subsequent physiological states, and consistent with persistent relationships among physiological response profiles.

The self-supervised learning process is governed by three complementary objectives. Physical consistency encourages two admissible views of the same physiological episode to produce compatible latent representations. Forward consistency exploits the chronological organization of repeated measurements by predicting the latent representation of a subsequent physiological state from the current state. Persistent relation preservation constrains pairwise relationships among physiological samples to remain stable across different admissible views. These objectives are jointly optimized through


$$L_{\text{SSL}}=\lambda_{\text{pc}}L_{\text{pc}}+\lambda_{\text{fc}}L_{\text{fc}}+\lambda_{\text{pr}}L_{\text{pr}},$$

where the three terms correspond to physical consistency, forward consistency, and persistent relational structure, respectively. Importantly, inflammatory-response class labels are not used in this self-supervised optimization stage.

Section 3.3 describes the architectural realization of these principles through three corresponding components: the Physical Consistency Encoder, the Forward Consistency Projector, and the Persistent Relation Extractor. The Physical Consistency Encoder transforms the multivariate physiological sequence into temporally organized latent representations. The Forward Consistency Projector operates on these latent states to estimate the representation expected at the next available physiological stage. The Persistent Relation Extractor constructs and preserves the pairwise relational structure among sequence-level physiological representations. The three components are therefore functionally distinct but jointly contribute to learning representations that capture sample-level consistency, temporal evolution, and population-level structure.

Section 3.4 further explains how these learned representations are used for latent dynamics modeling and future-state prediction. Given a latent physiological state **z***_i,t_*, the Forward Consistency Projector estimates the subsequent state


$${\hat{\text{z}}}_{i,t+1}=f_{\text{proj}}({\text{z}}_{i,t};\theta_{\text{proj}}),$$

and the same transition function can be recursively applied when multi-step prediction is required. This formulation treats the temporal ordering of physiological measurements as the self-supervisory signal and therefore does not require inflammatory class annotations for learning the latent transition dynamics.

After self-supervised representation learning, the learned sequence-level representation is used for downstream inflammatory-response pattern recognition. A lightweight classifier *g_ω_*maps the learned physiological representation to the predefined inflammatory-response categories. Ground-truth labels are introduced only during this downstream stage for classifier training and quantitative evaluation using Accuracy, Precision, Recall, and F1-score. When future inflammatory-state classification is required, the predicted future latent representation can similarly be supplied to the downstream classifier. The framework therefore explicitly separates label-free physiological representation learning from labeled inflammatory-response evaluation.

PhysicalForwardPersistent integrates representation consistency, temporal predictability, and persistent relational structure within a unified self-supervised framework. This design enables the model to exploit both cross-sectional and longitudinal information in physiological data while maintaining a clear distinction between self-supervised latent learning and downstream inflammatory-response classification. The following subsections provide the detailed mathematical formulation, architectural implementation, and temporal modeling strategy of the proposed approach.

### 3.2 Preliminaries

This subsection establishes the notation and self-supervised learning formulation used by the proposed PhysicalForwardPersistent model. Consistent with the dataset representation described in Section 4.1, the model operates on structured multivariate physiological observations rather than image data. Each sample corresponds to an exercise- or intervention-associated physiological sequence containing inflammationrelated biomarkers, basic physiological measurements, and exercise descriptors arranged according to their temporal relationship with the physical activity. Let the dataset be denoted by


$$D={\left\{{\text{X}}_{i}\right\}}_{i=1}^{N},$$

where *N* is the number of physiological samples and


$${\text{X}}_{i}=[{\text{x}}_{i,1},{\text{x}}_{i,2},\ldots ,{\text{x}}_{i,T_{i}}]\in {\mathbb{R}}^{T_{i}\times d}$$

represents the *i*-th multivariate physiological sequence. Here, *T_i_* denotes the number of available temporal observations and *d* is the number of input variables after preprocessing. Each observation ${\text{x}}_{i,t}\in {\mathbb{R}}^{d}$ may contain inflammation-related biomarkers, physiological measurements, and physical-activity descriptors such as exercise intensity, duration, and modality. Depending on the source dataset, the temporal observations may correspond to baseline, exercise, immediate post-exercise, and recovery stages, or to a more sparsely sampled pre/post-intervention structure.

For downstream evaluation, each complete sequence may additionally be associated with an inflammatory-response label


$$c_{i}\in C$$

where C denotes the set of predefined inflammatory-response categories described in Section 4.1. Importantly, *c_i_* is *not* used to optimize the representation-learning objective. The labels are introduced only during downstream evaluation for calculating classification metrics such as Accuracy, Precision, Recall, and F1-score. This distinction separates the label-free self-supervised representation-learning stage from the labeled evaluation stage. The PhysicalForwardPersistent framework learns a latent representation through three complementary principles: physical consistency, forward consistency, and persistent relational structure. Let


$$E_{\theta }:{\mathbb{R}}^{T_{i}\times d}\to {\mathbb{R}}^{T_{i}\times k}$$

denote the Physical Consistency Encoder parameterized by *θ*, where *k* is the latent dimensionality. The encoded sequence is


$${\text{Z}}_{i}=E_{\theta }({\text{X}}_{i})=[{\text{z}}_{i,1},\ldots ,{\text{z}}_{i,T_{i}}],\;\;{\text{z}}_{i,t}\in {\mathbb{R}}^{k}.$$

To construct a self-supervised learning signal without inflammatory class annotations, two physiologically admissible stochastic views of each input sequence are generated,


$${\tilde{\text{X}}}_{i}^{\left(1\right)}={ɡ}_{1}({\text{X}}_{i}),\;\;{\tilde{\text{X}}}_{i}^{\left(2\right)}={ɡ}_{2}({\text{X}}_{i}),$$

where ${ɡ}_{1},{ɡ}_{2}\in T$ are transformations selected to preserve the underlying physiological identity of the sample. These transformations operate on structured physiological variables and temporal observations and do not involve image-specific operations. Their implementation is detailed in Section 4.2.

Physical consistency.

The first learning principle requires representations derived from two admissible views of the same physiological sample to remain close, while representations originating from different samples remain distinguishable. Let


$${\text{h}}_{i}^{\left(v\right)}=\text{Pool }(E_{\theta }({\tilde{\text{X}}}_{i}^{\left(v\right)})),\;\;v\in \{1,2\},$$

denote a sequence-level latent representation obtained by temporal pooling. Physical consistency is implemented using a contrastive objective. For a mini-batch of *B* samples, the loss is defined as

(1) $$L_{\text{pc}}=-\frac{1}{B}\overset{B}{\underset{i=1}{\sum }}\text{log}\frac{exp\;(sim\;({\text{h}}_{i}^{\left(1\right)},{\text{h}}_{i}^{\left(2\right)})/\tau )}{\sum_{j=1}^{B}exp\;(sim\;({\text{h}}_{i}^{\left(1\right)},{\text{h}}_{j}^{\left(2\right)})/\tau )},$$

where


$$\text{sim }(\text{u},\text{v})=\frac{{\text{u}}^{⊤}\text{v}}{\|\text{u}{\|}_{2}\|v{\|}_{2}}$$

is cosine similarity and *τ >* 0 is a temperature parameter. Equation 1 provides an explicit self-supervised mechanism for preserving physiologically meaningful information under admissible perturbations without requiring inflammatory-response labels.

Forward consistency. The second principle exploits the temporal organization of the physiological observations. Let


$$P_{\phi }:{\mathbb{R}}^{k}\to {\mathbb{R}}^{k}$$

denote the Forward Consistency Projector parameterized by *ϕ*. Given the latent state z*_i,t_*, the projector estimates the representation expected at the subsequent physiological stage,


$${\hat{z}}_{i,t+1}=P_{\phi }(z_{i,t}).$$

The corresponding forward-consistency objective is

(2) $${ℒ}_{\text{fc}}=\frac{1}{\sum_{i=1}^{B}\left(T_{i}-1\right)}\sum_{i=1}^{B}\sum_{t=1}^{T_{i}-1}{\left\|P_{\phi }\left({\text{z}}_{i,t}\right)-\text{sg }({\text{z}}_{i,t+1})\right\|}_{2}^{2},$$

where sg(·) denotes the stop-gradient operator. In contrast to a supervised future-state predictor, Equation 2 uses the subsequent observation itself as the learning target and therefore does not require an externally assigned inflammatory class label. This term encourages the latent representation to encode temporal changes associated with exercise and recovery rather than only static physiological characteristics.

Persistent relational structure. The third principle is designed to preserve stable relationships among physiological representations. For two latent representations h*_i_* and h*_j_*, their relation is quantified using a Gaussian similarity function, Equation 3

(3) $$R({\text{h}}_{i},{\text{h}}_{j})=\text{exp }(-\frac{\|{\text{h}}_{i}-{\text{h}}_{j}{\|}_{2}^{2}}{2\sigma^{2}}),$$

where *σ >* 0 controls the sensitivity of the relation measure. Rather than directly minimizing the relation values, as would incorrectly encourage mutually similar samples to become dissimilar, the Persistent Relation Extractor seeks to maintain the relational structure across admissible views. Let


$$R_{ij}^{\left(v\right)}=R({\text{h}}_{i}^{\left(v\right)},{\text{h}}_{j}^{\left(v\right)}),\;\;v\in \{1,2\}.$$

The persistent-relation loss is therefore defined as

$L_{\text{pr}}=\frac{1}{B(B-1)}\underset{i\neq j}{\sum_{i,j=1}^{B}}{\left(R_{ij}^{\left(1\right)}-R_{ij}^{\left(2\right)}\right)}^{2}.$ This objective encourages pairwise physiological relationships that are stable in one admissible representation of the data to remain stable in another, thereby reducing sensitivity to nuisance variation while retaining the relative organization of samples in the latent space.

Composite self-supervised objective. The three learning principles address complementary aspects of the problem. Physical consistency promotes invariance to physiologically admissible perturbations, forward consistency captures temporal evolution across exercise and recovery stages, and persistent relation preservation maintains stable inter-sample structure. The complete self-supervised objective is consequently formulated as

(4) $$L_{\text{SSL}}=\lambda_{\text{pc}}L_{\text{pc}}+\lambda_{\text{fc}}L_{\text{fc}}+\lambda_{\text{pr}}L_{\text{pr}},$$

where $\lambda_{\text{pc}},\lambda_{\text{fc}},\lambda_{\text{pr}}>0$ control the relative contribution of the three objectives. Their experimental settings are reported in Section 4.2.

The formulation in Equation 4 is motivated by the structure of the physiological task rather than by an arbitrary combination of regularization terms. Exercise-associated inflammatory observations contain three forms of information that should be retained simultaneously: measurements from the same physiological episode should remain representation-consistent under non-destructive perturbations; consecutive measurements should encode the directional evolution from exercise toward subsequent inflammatory or recovery states; and stable relationships among physiological response profiles should be preserved despite variations in individual measurements. The three objective terms explicitly correspond to these requirements. After self-supervised optimization, the learned representation can be used for downstream inflammatory-response pattern recognition. The ground-truth inflammatory categories introduced in Section 4.1 are used only at this downstream stage and are never included in L_SSL_. Therefore, the reported classification performance evaluates the physiological information captured by the selfsupervised representation rather than implying that inflammatory labels were required to construct that representation. This formulation provides the mathematical foundation for the three modules of PhysicalForwardPersistent. Section 3.3 specifies their concrete architectural realization, while Section 3.4 further describes how the learned latent dynamics are used for future-state modeling and integrated optimization.

### 3.3 PhysicalForwardPersistent: a novel model for inflammatory response pattern recognition

Building upon the self-supervised formulation introduced in Section 3.2, we propose PhysicalForwardPersistent, a unified framework for learning physiologically informative representations from multivariate inflammatory-response sequences associated with physical education interventions. The model is organized around three complementary components: Physical Consistency Encoding, Forward Consistency Projection, and Persistent Relation Extraction. Rather than treating these components as isolated processing blocks, they are jointly optimized through the composite self-supervised objective in **Equation 4**. Each component addresses a different characteristic of exercise-associated physiological data: invariance to non-destructive perturbations, temporal evolution across exercise and recovery stages, and preservation of stable relationships among physiological response profiles.

As illustrated in **Figure 1**, PhysicalForwardPersistent integrates physical consistency encoding, forward consistency projection, and persistent relation extraction within a unified self-supervised framework. These complementary components jointly encourage perturbation-invariant representation learning, temporal-state predictability, and preservation of population-level physiological relations.

**Figure 1:**
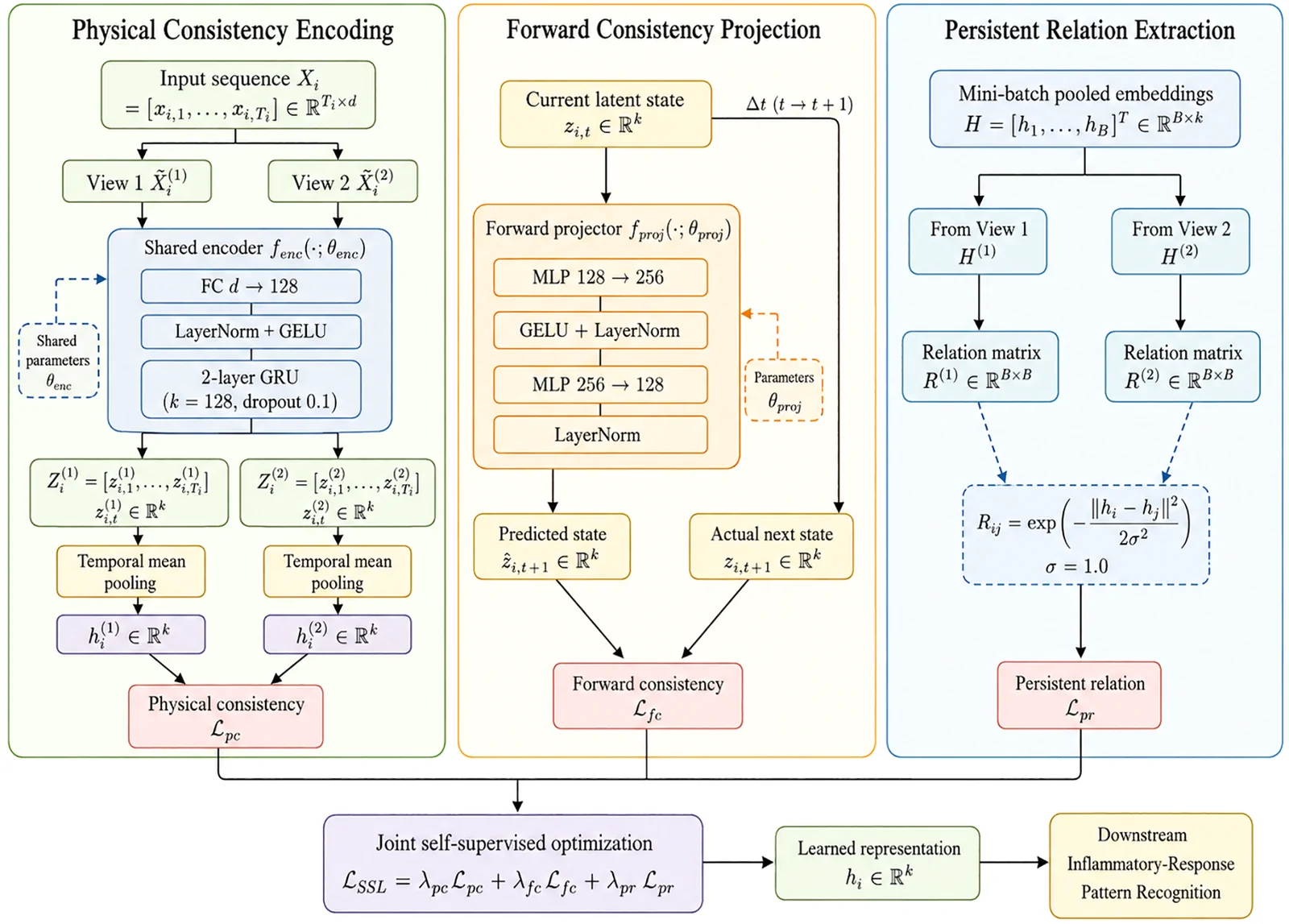
Overview of the proposed PhysicalForwardPersistent framework. Two admissible views of each multivariate physiological sequence are processed by a shared physical consistency encoder to obtain temporally organized latent representations and pooled sequence-level embeddings. The Forward Consistency Projector predicts the subsequent latent physiological state from the current state, while the Persistent Relation Extractor preserves pairwise relationships among physiological response profiles across different self-supervised views. The physical-consistency, forward-consistency, and persistent-relation objectives are jointly optimized to learn physiologically informative representations for downstream inflammatory-response pattern recognition.

Physical Consistency Encoding. The Physical Consistency Encoder transforms each multivariate physiological sequence into a temporally organized latent representation. For the *i*-th sample,


$${\text{X}}_{\text{i}}=[{\text{x}}_{\text{i},1},\ldots ,{\text{x}}_{i,{\text{T}}_{\text{i}}}]\in {\mathbb{R}}^{T_{i}\times d},$$

where *T_i_* is the number of available physiological observations and *d* is the input feature dimension after preprocessing. The encoder is defined as


$$f_{\text{enc}}:{\mathbb{R}}^{T_{i}\times d}\to {\mathbb{R}}^{T_{i}\times k},$$

and produces the latent sequence Equation 5

(5) $${\text{Z}}_{i}=f_{\text{enc}}({\text{X}}_{i};\theta_{\text{enc}})=[{\text{z}}_{i,1},\ldots ,{\text{z}}_{i,T_{i}}],\;\;{\text{z}}_{i,t}\in {\mathbb{R}}^{k},$$

where $\theta_{\text{enc}}$ denotes the learnable encoder parameters.

In the implementation, each physiological observation is first projected from the original *d*-dimensional feature space to a 128-dimensional hidden representation using a fully connected input layer, followed by Layer Normalization and a GELU activation function. The projected sequence is subsequently processed by a two-layer gated recurrent unit (GRU) network with a hidden dimension of 128 and a dropout rate of 0.1. The output dimensionality is therefore set to *k* = 128. A recurrent temporal encoder is adopted because the input consists of ordered physiological observations rather than spatial image features, and the same architecture can accommodate both sparsely sampled pre/post-intervention records and more densely sampled exercise–recovery sequences.

Given two admissible views ${\tilde{\text{X}}}_{i}^{\left(1\right)}$ and ${\tilde{\text{X}}}_{i}^{\left(2\right)}$ of the same physiological episode, the two sequences are processed by the same encoder with shared parameters. A sequence-level representation is obtained through temporal mean pooling,


$${\text{h}}_{i}^{\left(v\right)}=\frac{1}{T_{i}}\sum_{t=1}^{T_{i}}{\text{z}}_{i,t}^{\left(v\right)},\;\;v\in \{1,2\}.$$

These representations are used by the physical-consistency objective in Equation (1). Weight sharing ensures that the agreement between the two views arises from the learned representation rather than from separate view-specific encoders. The resulting latent space is therefore encouraged to preserve inflammatory and exercise-related information that remains stable under physiologically admissible perturbations while suppressing nuisance variation introduced by measurement noise or incomplete observations.

Forward Consistency Projection. The Forward Consistency Projector explicitly models the directional evolution of the latent physiological state. Instead of projecting the complete sequence into an unrelated feature space, the projector operates on each latent state and predicts the representation corresponding to the next available physiological stage. For an encoded state **z***_i,t_*, the forward projection is defined as Equation 6

(6) $${\hat{\text{z}}}_{i,t+1}=f_{\text{proj}}({\text{z}}_{i,t};\theta_{\text{proj}}),\;\;t=1,\ldots ,T_{i}-1,$$

where $f_{\text{proj}}:{\mathbb{R}}^{k}\to {\mathbb{R}}^{k}$ and $\theta_{\text{proj}}$ denotes its learnable parameters.

The projection head consists of a two-layer multilayer perceptron with dimensions *k* → 2*k* → *k*. With *k* = 128, this corresponds to hidden dimensions 128 → 256 → 128. A GELU nonlinearity is applied after the first linear transformation, and Layer Normalization is applied to the projected output. This structure provides sufficient capacity to model nonlinear physiological transitions while keeping the dimensionality of the predicted state identical to that of the target latent state.

The projector is optimized using the forward-consistency objective in Equation 2, which compares ${\hat{\text{z}}}_{i,t+1}$ with the latent representation of the actually observed subsequent physiological stage. Consequently, no manually assigned inflammatory-response class is required to create the prediction target. The temporal organization of the physiological measurements itself provides the supervisory signal. For datasets containing only pre- and post-intervention observations, a single transition is modeled for each sequence. For datasets containing baseline, exercise, post-exercise, and recovery observations, the same projector is shared across all consecutive transitions.

This formulation is particularly important for inflammatory-response modeling because the physiological consequence of exercise is inherently dynamic. The representation at one time point should not only describe the current biomarker profile but should also retain information that is predictive of its subsequent evolution. Forward consistency therefore complements the invariance objective of the encoder by introducing an explicit temporal constraint.

Persistent Relation Extraction. Although physical and forward consistency operate primarily at the individual-sample level, inflammatory responses also exhibit relational structure across participants and exercise episodes. The Persistent Relation Extractor is introduced to preserve this inter-sample organization during self-supervised learning.

For a mini-batch of *B* physiological sequences, the temporally pooled latent representations are collected as


$$\text{H}={\left[{\text{h}}_{1},{\text{h}}_{2},\ldots ,{\text{h}}_{B}\right]}^{⊤}\in {\mathbb{R}}^{B\times k}.$$

The extractor constructs the corresponding pairwise relation matrix

(7) $$\text{R}=f_{\text{extr}}(\text{H};\sigma ),\;\;R_{ij}=\text{exp }(-\frac{\|{\text{h}}_{i}-{\text{h}}_{j}{\|}_{2}^{2}}{2\sigma^{2}}),$$

where $\text{R}\in {\mathbb{R}}^{B\times B}$ and σ>0 controls the sensitivity of the relational similarity. In the configuration, *σ* is initialized to 1.0 and selected using the validation partition.

Unlike a conventional classification head, the relation extractor does not require inflammatory-response labels. Instead, it evaluates whether the relative organization of samples is preserved when the same physiological observations are subjected to different admissible perturbations. Relation matrices R^(1)^ and R^(2)^ are constructed from the two self-supervised views, and their consistency is enforced by the persistentrelation objective defined in Section 3.2. This formulation avoids directly minimizing pairwise similarity values, which would undesirably push physiologically related samples apart. Instead, the model preserves the relational geometry that remains stable across different representations of the same mini-batch.

The relation extractor is deliberately lightweight and kernel-based rather than implemented as an additional deep network. This design makes its role distinguishable from that of the encoder: the encoder learns nonlinear physiological features, whereas the extractor regularizes the organization of those features at the population level. Consequently, improvements associated with this component can be attributed to relational preservation rather than simply to an increase in network depth or parameter count.

Joint learning and representation output.During self-supervised training, the Physical Consistency Encoder, Forward Consistency Projector, and Persistent Relation Extractor operate jointly. For every minibatch, two admissible views of each physiological sequence are encoded using shared encoder parameters. The resulting latent representations contribute to the physical-consistency loss, consecutive latent states are provided to the Forward Consistency Projector to calculate the forward-consistency loss, and the pooled sequence representations are used to construct relation matrices for the persistent-relation loss. The overall parameters are optimized according to


$$L_{\text{SSL}}=\lambda_{\text{pc}}L_{\text{pc}}+\lambda_{\text{fc}}L_{\text{fc}}+\lambda_{\text{pr}}L_{\text{pr}},$$

as defined in Equation 4.

After self-supervised training, the sequence-level representation produced by the Physical Consistency Encoder serves as the principal representation for downstream inflammatory-response pattern recognition. The inflammatory-response labels defined in Section 4.1 are introduced only in this downstream stage and are not used to optimize the self-supervised objective. The Forward Consistency Projector and Persistent Relation Extractor therefore act as complementary self-supervised constraints that shape the physiological representation learned by the encoder.

This division of roles is important for interpreting the proposed framework. Physical Consistency Encoding preserves information that is stable under non-destructive physiological perturbations; Forward Consistency Projection encourages the representation to retain predictive information about temporal evolution; and Persistent Relation Extraction preserves population-level relationships among inflammatoryresponse profiles. Their joint optimization provides a representation that simultaneously captures samplelevel consistency, temporal dynamics, and relational structure, forming the basis for the future-state modeling strategy described in Section 3.4.

### 3.4 Latent dynamics modeling and future state prediction

Based on the representation-learning architecture described in Section 3.3, we further formulate how PhysicalForwardPersistent models the temporal evolution of exercise-associated inflammatory responses. The purpose of this strategy is not to introduce an additional learning objective independent of the self-supervised framework, but to explicitly describe how the latent representations learned by the three components are used to characterize physiological transitions and predict subsequent inflammatory states. In particular, latent dynamics are modeled through the Physical Consistency Encoder and Forward Consistency Projector, while the Persistent Relation Extractor regularizes the stability of inter-sample physiological relationships throughout representation learning.

The latent-dynamics and future-state prediction strategy is summarized in **Figure 2**. The learned latent representation is organized according to physiological time, and the same Forward Consistency Projector is recursively applied to characterize both immediate and multi-step physiological evolution. Relation preservation and the unified self-supervised objective jointly regularize the learned dynamics before downstream inflammatory-state prediction.

**Figure 2:**
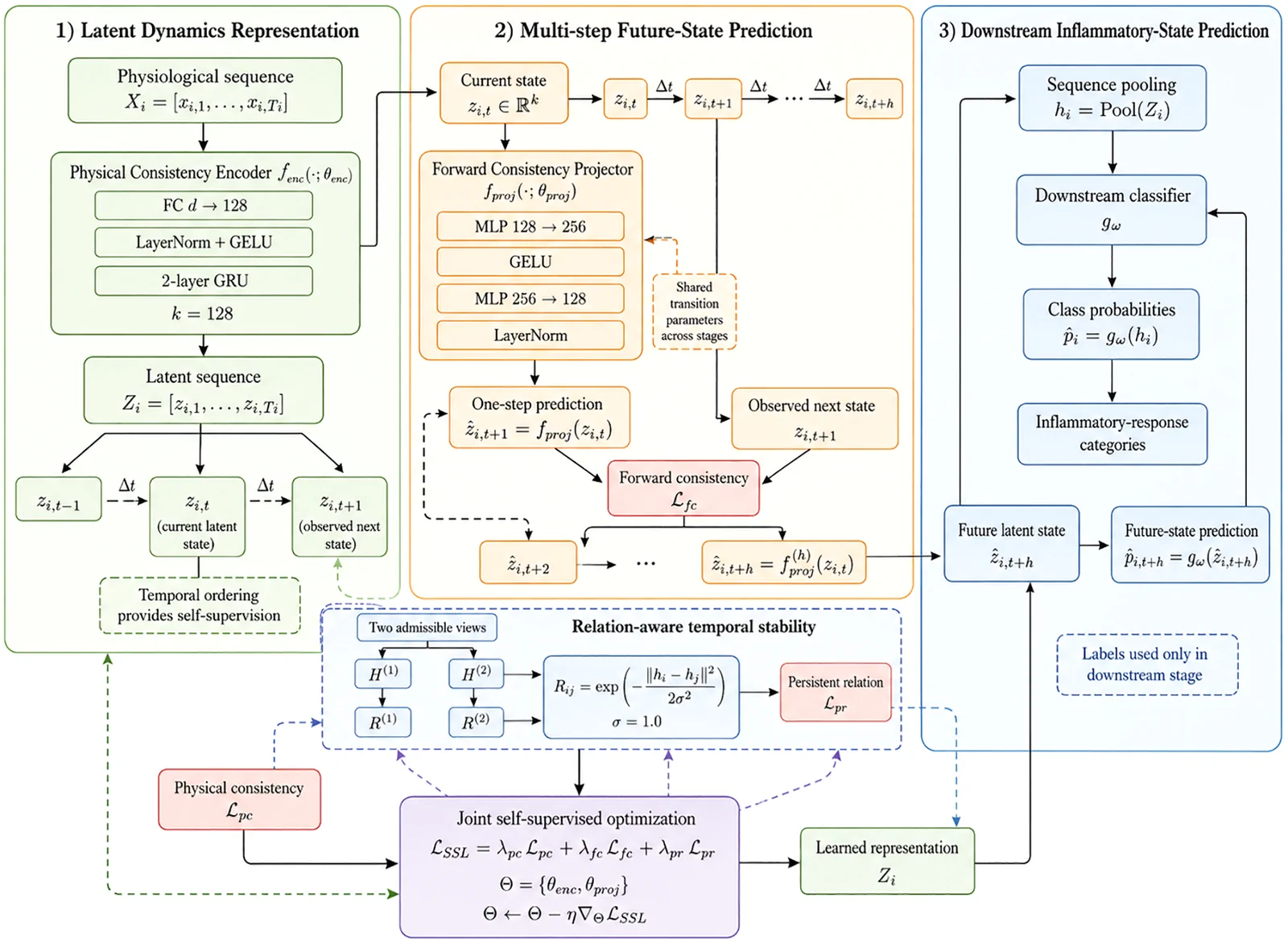
Latent dynamics modeling and future-state prediction in PhysicalForwardPersistent. The physical consistency encoder maps an ordered physiological sequence into a sequence of latent states representing successive exercise- and recovery-associated physiological conditions. The Forward Consistency Projector learns the transition from the current latent state to the subsequent state and can be recursively applied for multi-step future-state prediction using shared transition parameters. Persistent relation preservation maintains stable inter-sample organization during self-supervised learning, while the unified objective jointly optimizes physical consistency, temporal predictability, and relational stability. After representation learning, pooled or predicted future latent states are supplied to a lightweight downstream classifier for inflammatory-state recognition.

Latent dynamics representation. For the *i*-th physiological sequence,


$${\text{X}}_{i}=[{\text{x}}_{i,1},\ldots ,{\text{x}}_{i,T_{i}}],$$

the Physical Consistency Encoder produces the temporally organized latent sequence


$${\text{Z}}_{i}=[{\text{z}}_{i,1},\ldots ,{\text{z}}_{i,T_{i}}],\;\;{\text{z}}_{i,t}\in {\mathbb{R}}^{k}.$$

Each latent state summarizes the physiological condition observed at a particular stage relative to the exercise or physical education intervention. Depending on the dataset, these stages may include baseline, exercise, immediate post-exercise, and recovery measurements, or a simpler pre/post-intervention sequence.

The latent dynamics are represented by the transition from **z***_i,t_*to **z***_i,t_*_+1_. Rather than assuming a fixed linear change between consecutive stages, the Forward Consistency Projector learns a nonlinear transition function, Equation 8

(8) $${\hat{\text{z}}}_{i,t+1}=f_{\text{proj}}({\text{z}}_{i,t};\theta_{\text{proj}}),$$

where ${\hat{\text{z}}}_{i,t+1}$ denotes the predicted latent representation of the subsequent physiological state. The actually observed next state, **z***_i,t_*_;+1_, provides the self-supervised prediction target through the forward-consistency loss defined in Equation 2.

This formulation allows temporal information to be learned directly from the ordering of physiological observations. Therefore, the model does not require an externally assigned inflammatory-response class to learn the transition from one physiological stage to the next. Instead, the chronological structure of the measurements provides the supervisory signal.

Multi-step future-state prediction. For sequences containing more than two observations, the learned transition function can be recursively applied to estimate physiological states beyond the immediately subsequent observation. Starting from the observed latent state z*_i,t_*, an *h*-step prediction is obtained as

(9) $${\hat{\text{z}}}_{i,t+h}=f_{\text{proj}}^{\left(h\right)}({\text{z}}_{i,t}),\;\;h\geq 1,$$

where $f_{\text{proj}}^{\left(h\right)}$ denotes *h* recursive applications of the same Forward Consistency Projector.


$${\hat{\text{z}}}_{i,t+1}=f_{\text{proj}}({\text{z}}_{i,t})$$

and


$${\hat{\text{z}}}_{i,t+h}=f_{\text{proj}}({\hat{\text{z}}}_{i,t+h-1}),\;\;h>1.$$

The use of shared transition parameters across temporal stages limits unnecessary model complexity and encourages the learned dynamics to reflect recurrent physiological progression rather than stage-specific memorization. For datasets containing only pre- and post-intervention measurements, the framework reduces naturally to one-step latent-state prediction.

During training, one-step prediction remains the primary temporal learning signal because the corresponding future latent state is directly observed. When multiple subsequent observations are available, prediction quality across longer horizons can additionally be evaluated by comparing recursively predicted states with their corresponding encoded observations. This formulation provides a consistent mechanism for examining whether the learned representation captures not only the current inflammatory profile but also its subsequent exercise- and recovery-associated evolution.

Relation-aware temporal stability. Temporal prediction alone does not guarantee that the organization of different physiological response profiles remains stable. For example, small measurement perturbations could alter the relative arrangement of samples even when their underlying inflammatory-response characteristics remain similar. The Persistent Relation Extractor therefore complements forward prediction by preserving pairwise relationships among sequence-level latent representations.

Let R^(1)^ and R^(2)^ denote the relation matrices constructed from two admissible views of the same minibatch, as defined in Section 3.2. Their discrepancy is minimized through $L_{\text{pr}}$. Consequently, the model is encouraged to preserve population-level relational structure while simultaneously learning temporal state transitions.

The roles of forward consistency and persistent relation preservation are therefore distinct but complementary. Forward consistency constrains the direction of within-sequence physiological evolution, whereas persistent relation preservation constrains the stability of between-sequence organization. Together, they reduce the risk that temporal prediction is achieved at the expense of physiologically meaningful relational structure.

Self-supervised optimization of latent dynamics. The latent-dynamics strategy is optimized using the same unified self-supervised objective introduced in Section 3.2, Equation 10

(10) $$L_{\text{SSL}}=\lambda_{\text{pc}}L_{\text{pc}}+\lambda_{\text{fc}}L_{\text{fc}}+\lambda_{\text{pr}}L_{\text{pr}}.$$

No additional independent objective such as $L_{\text{latent}}+L_{\text{temporal}}$ is introduced. This avoids defining multiple competing optimization criteria for the same model. Instead, the three terms in L_SSL_ jointly govern representation invariance, temporal prediction, and relational stability.

Let

$\text{Θ}=\{\theta_{\text{enc}},\theta_{\text{proj}}\}$ denote the learnable parameters of the encoder and projector. Because the Persistent Relation Extractor is implemented through the relation function defined in Equation 7, its sensitivity parameter *σ* is treated according to the configuration described in Section 4.2. The learnable model parameters are updated by gradient-based optimization as Equation 11

(11) $$\text{Θ}\leftarrow \text{Θ}-\eta \nabla_{\text{Θ}}L_{\text{SSL}},$$

where *η* denotes the learning rate. Since $L_{\text{pc}}$, $L_{\text{fc}}$, and $L_{\text{pr}}$ depend on shared latent representations, gradients from the three objectives jointly shape the encoder. The resulting representation is therefore optimized simultaneously for physical consistency, temporal predictability, and relational stability.

Downstream inflammatory-state prediction. After self-supervised representation learning, the learned latent features are used for downstream inflammatory-response pattern recognition. For each complete physiological sequence, a sequence-level representation is obtained by pooling the encoded latent states,


$${\text{h}}_{i}=\text{Pool }({\text{Z}}_{i}).$$

A lightweight downstream classifier $g_{\omega }$maps this representation to the predefined inflammatory-response categories, Equation 12

(12) $${\hat{\text{p}}}_{i}=g_{\omega }({\text{h}}_{i}),$$

where ${\hat{p}}_{i}$ denotes the predicted class-probability vector. The ground-truth inflammatory-response labels described in Section 4.1 are used at this stage for downstream training and evaluation, but they are not used to construct the self-supervised representations.

When prediction of a subsequent inflammatory state is required, the predicted future latent representation can instead be supplied to the same downstream prediction head,


$${\hat{\text{p}}}_{i,t+h}=g_{\omega }({\hat{\text{z}}}_{i,t+h})$$

thereby separating two related tasks: the Forward Consistency Projector learns the physiological transition in latent space without inflammatory class labels, whereas the downstream classifier translates the resulting latent representation into an interpretable inflammatory-response category.

This distinction is essential to the proposed framework. Self-supervision is used to learn the structure and temporal evolution of physiological responses, while labeled information is reserved for downstream inflammatory-state recognition and quantitative evaluation.

Integrated interpretation of the strategy. The resulting latent-dynamics framework provides a unified description of how PhysicalForwardPersistent processes exercise-associated physiological information. The Physical Consistency Encoder constructs representations that are stable under physiologically admissible perturbations. The Forward Consistency Projector models the directional evolution of these representations across exercise and recovery stages. The Persistent Relation Extractor preserves stable relationships among different physiological response profiles. The downstream classifier converts the learned representations into inflammatory-response categories for evaluation.

Accordingly, future-state prediction in PhysicalForwardPersistent should be interpreted as prediction of the evolution of the latent physiological state followed, when required, by downstream inflammatorystate classification. This formulation avoids conflating label-free self-supervised temporal learning with labeled classification and provides a coherent connection between the mathematical formulation, model architecture, and experimental evaluation.

## 4 Experimental setup

### 4.1 Dataset

To provide a transparent and reproducible description of the data used in this study, we summarize the main characteristics of the four datasets in **Table 1**. Although the datasets were collected under different exercise or intervention protocols, they were represented using a common physiological data format before being provided to the proposed PhysicalForwardPersistent model and the competing methods. Each sample was represented as a multivariate physiological sequence consisting of inflammation-related biomarkers, basic physiological measurements, and exercise-related descriptors arranged according to their temporal order relative to the exercise intervention. Thus, the input to the learning algorithms consisted of structured physiological data rather than images. When repeated measurements were available, the observations were ordered from the pre-exercise baseline to the exercise and recovery phases. For datasets containing only pre- and post-intervention measurements, the two observations were retained as a short temporal sequence.

**Table 1:** Summary of the datasets used in the experiments.

Dataset	Participants	Samples	Feature dim.	Time points	Low	Moderate	High
Physical Activity Inflammation Response	240	1,920	18	4	672	650	598
Exercise Induced Cytokine Patterns	180	1,440	14	5	500	490	450
Self-Supervised Learning Inflammation	320	2,560	24	4	890	860	810
Physical Education Intervention Biomarker	260	2,080	20	2	720	700	660

The Physical Activity Inflammation Response Dataset Quiroga et al. (2020) contains physiological and biochemical measurements collected from participants performing physical activities with different intensities and durations. In the present experimental configuration, the dataset contains 240 participants and 1,920 exercise-associated samples. The participant cohort has an approximately balanced sex distribution and an age range of approximately 18–35 years. Each sample contains 18 physiological and exerciserelated variables, including circulating inflammatory markers, heart rate-derived measurements, and descriptors of exercise duration, intensity, and modality. Measurements obtained at baseline, during exercise, immediately after exercise, and during recovery are organized chronologically to form a fourstep physiological sequence. This representation enables the model to capture both the magnitude of the inflammatory response and its temporal evolution following physical activity. The Exercise Induced Cytokine Patterns Dataset Islam et al. (2024) specifically characterizes exercise-associated cytokine dynamics. The dataset configuration contains 180 participants and 1,440 samples, with 14 variables measured across five temporal stages covering the pre-exercise, exercise, immediate post-exercise, and recovery periods. The principal inflammatory variables include cytokine-related measurements such as IL-6, TNF-*α*, IL-1*β*, and anti-inflammatory cytokine activity, together with exercise-intensity and basic physiological variables. These repeated measurements make the dataset particularly suitable for evaluating whether the proposed model can distinguish transient exercise-induced cytokine elevations from more persistent inflammatory response patterns. The Self-Supervised Learning Inflammation Dataset Margapuri (2025) contains heterogeneous inflammation-related physiological observations originating from multiple experimental or observational settings. For the present experiments, the dataset contains 320 participants and 2,560 samples, with each sample described by 24 variables. The feature set includes inflammatory biomarkers, physiological measurements, and available contextual variables describing the physical-activity condition. Where repeated observations are available, measurements are temporally aligned relative to the intervention or activity onset. The relatively heterogeneous structure of this dataset provides a useful setting for examining the ability of self-supervised representation learning to extract transferable patterns from physiological data without requiring inflammatory class annotations during representation learning.

The Physical Education Intervention Biomarker Dataset Park et al. (2024) focuses on biomarker changes associated with structured physical education interventions. The experimental subset consists of 260 participants and 2,080 samples characterized by 20 variables. The variables include markers related to inflammation, cardiovascular status, metabolic function, and physical-education intervention characteristics. Because the original intervention structure primarily consists of measurements obtained before and after the physical education program, each sample is represented by a two-stage temporal sequence. This dataset therefore complements the more densely sampled exercise datasets by evaluating whether the proposed method remains effective when only relatively sparse longitudinal measurements are available.

#### 4.1.1 Participant selection and data quality control

Across the four datasets, participants were included when they completed the corresponding exercise or physical education protocol and had at least one valid baseline measurement together with the required post-exercise or post-intervention inflammatory assessment. Records were excluded when the exercise protocol was incomplete, when more than 20% of the required variables were missing, or when physiological measurements contained substantial acquisition artifacts. Where such information was available, participants presenting acute infection or taking medication expected to substantially alter systemic inflammatory responses were excluded according to the original study protocols. Demographic variables were retained for dataset characterization but were not used as direct inflammatory labels. Demographic information was reported according to its availability in the source datasets. Age and sex distributions are summarized where these variables were available in the original records; for datasets in which complete demographic metadata were not provided, no demographic values were imputed or inferred. Demographic variables were used only for cohort characterization and were not used to define the inflammatory-response ground truth.

#### 4.1.2 Input features and preprocessing

The input variables were grouped into three categories: inflammation-related biomarkers, including cytokine and acute-phase response measurements; physiological variables, such as heart rate and other available vital-sign measurements; and exercise-related descriptors, including exercise type, duration, and intensity. The exact feature subset varied slightly across datasets according to data availability, as reflected by the feature dimensions reported in **Table 1**. Missing continuous variables were imputed using the median estimated exclusively from the training set, whereas categorical variables were imputed using the most frequent training-set category. Continuous variables were subsequently standardized using z-score normalization Equation 13

(13) $${\tilde{x}}_{j}=\frac{x_{j}-\mu_{j}^{train}}{\sigma_{j}^{train}},$$

where $\mu_{j}^{\text{train}}$ and $\sigma_{j}^{\text{train}}$ denote the mean and standard deviation of feature *j* calculated from the training partition only. Exercise modality and other categorical descriptors were converted to one-hot representations when required. All preprocessing parameters were estimated using the training data and subsequently applied unchanged to the validation and test sets to avoid information leakage. For longitudinal records, measurements were ordered according to their temporal relationship with the exercise intervention. When the sampling schedules differed between participants, observations were aligned to the predefined intervention phases rather than treated as image-like inputs. No image-specific preprocessing or image-specific augmentation was applied

#### 4.1.3 Ground-truth definition

An important distinction was made between self-supervised representation learning and downstream evaluation. During the self-supervised learning stage, inflammatory class labels were not provided to the encoder. Instead, the model learned latent representations by exploiting the internal consistency and temporal relationships of the physiological observations. Ground-truth labels were used only during downstream evaluation to quantify pattern-recognition performance using Accuracy, Precision, Recall, and F1-score. For the experimental protocol, inflammatory responses were grouped into three categorieslow, moderate, and high response-according to a composite inflammatory response index derived from changes relative to the pre-exercise or pre-intervention baseline. The index combines standardized changes in representative inflammatory biomarkers, including IL-6, TNF-*α*, IL-1*β*, CRP, and available antiinflammatory cytokine measurements. When a biomarker was unavailable in a particular dataset, the index was calculated using the subset of predefined markers available for that dataset. For participant *i*, the change in each available inflammatory biomarker *m* was first calculated relative to the corresponding pre-exercise or pre-intervention baseline and standardized using statistics estimated exclusively from the training partition. The composite inflammatory-response index was subsequently defined as the mean standardized change across the *M_i_*predefined inflammatory markers available for participant *i*, Equation 14

(14) $$S_{i}=\frac{1}{M_{i}}\sum_{m=1}^{M_{i}}z(\text{Δ}b_{i,m}),$$

where $\text{Δ}b_{i,m}$ denotes the baseline-relative change in biomarker *m*, and *z*(·) denotes standardization using the mean and standard deviation estimated from the training partition. For anti-inflammatory markers whose increase represents a counter-regulatory response, the sign of $\text{Δ}b_{i,m}$ was oriented consistently before aggregation so that a larger *S_i_* represented a stronger overall inflammatory-response pattern. The low-, moderate-, and high-response categories were defined according to the lower and upper tertiles of *S_i_* estimated exclusively from the training partition. The resulting cut-off values were subsequently applied unchanged to the validation and test partitions to avoid information leakage.

#### 4.1.4 Training, validation, and test partitioning

To prevent observations from the same participant from appearing in different subsets, all datasets were divided at the participant level rather than at the individual-record level. A 70%/15%/15% training, validation, and test split was used for all four datasets. This resulted in 168/36/36 participants for the Physical Activity Inflammation Response Dataset, 126/27/27 participants for the Exercise Induced Cytokine Patterns Dataset, 224/48/48 participants for the Self-Supervised Learning Inflammation Dataset, and 182/39/39 participants for the Physical Education Intervention Biomarker Dataset. The corresponding physiological samples inherited the partition of their associated participants, thereby eliminating participantlevel leakage between training and evaluation data.

The same data partitions and the same preprocessed physiological input variables were used for PhysicalForwardPersistent and all SOTA comparison methods. Therefore, differences in predictive performance cannot be attributed to different training/test partitions or to access to different physiological information. During self-supervised training, the proposed model did not use the inflammatory-response class labels. The labels were introduced only when evaluating the learned representations and calculating the downstream classification metrics.

### 4.2 Experimental details

All experiments were implemented using Python 3.8 and PyTorch 1.9 with CUDA 11.2. Training was conducted on NVIDIA Tesla V100 GPUs with 32 GB memory. To ensure consistency across experiments, the proposed PhysicalForwardPersistent model and all comparison methods were trained and evaluated using the same participant-level training, validation, and test partitions described in Section 4.1. All preprocessing parameters were estimated exclusively from the training partition and subsequently applied without modification to the validation and test sets.

The experimental protocol operates directly on the structured multivariate physiological sequences defined in Sections 3.2 and 3.3. No ImageNet pre-training, image cropping, image flipping, color transformation, or other image-specific preprocessing was used. The same standardized physiological variables were provided to PhysicalForwardPersistent and the comparison methods to ensure that performance differences were attributable to the learning algorithms rather than to different input information.

#### 4.2.1 Model configuration

The implementation follows the architecture introduced in Section 3.3. The Physical Consistency Encoder first projects each physiological observation into a 128-dimensional hidden space and subsequently processes the temporally ordered sequence using a two-layer GRU with a hidden dimension of 128. Layer Normalization and GELU activation are applied after the input projection, and a dropout rate of 0.1 is used between recurrent layers. Consequently, the latent dimensionality is set to *k* = 128. The Forward Consistency Projector is implemented as a two-layer multilayer perceptron with dimensions 128 → 256 → 128, followed by GELU activation and Layer Normalization. The Persistent Relation Extractor uses the Gaussian relation function defined in Equation 7, with the initial relation-scale parameter set to *σ* = 1.0. For the physical-consistency objective, cosine similarity is calculated with a temperature parameter *τ* = 0.2. The weights of the three self-supervised objective terms are set to


$$\lambda_{\text{pc}}=1.0,\;\;\lambda_{\text{fc}}=0.5,\;\;\lambda_{\text{pr}}=0.1,$$

respectively. These hyperparameters were selected using the validation partition and were kept fixed when evaluating the test set. The main implementation settings are summarized in **Table 2**.

**Table 2:** Main implementation and training settings of PhysicalForwardPersistent.

Configuration	Setting
Latent dimension *k*	128
Encoder	2-layer GRU
GRU hidden dimension	128
Encoder dropout	0.1
Projection head	128–256–128 MLP
Activation function	GELU
Relation parameter *σ*	1.0
Contrastive temperature *τ*	0.2
*λ*pc	1.0
*λ*fc	0.5
*λ*pr	0.1
Batch size	64
SSL learning rate	1 × 10^−3^
Downstream learning rate	1 × 10^−4^
Weight decay	1 × 10^−4^
SSL training epochs	100
Downstream training epochs	50
Warm-up epochs	5
Optimizer	AdamW

#### 4.2.2 Physiological data augmentation

Because the experimental inputs consist of structured physiological observations rather than images, the augmentation strategy was designed specifically for multivariate biomarker and physiological data. During self-supervised training, two independently perturbed views ${\tilde{\text{X}}}_{i}^{\left(1\right)}$ and ${\tilde{\text{X}}}_{i}^{\left(2\right)}$ were generated from each physiological sequence.

The admissible transformations included small-magnitude Gaussian perturbation of standardized continuous variables, random masking of a limited proportion of continuous features, and temporalpoint masking for sequences containing more than two observations. Gaussian noise with a standard deviation of 0.05 in the normalized feature space was applied with a probability of 0.5, while up to 10% of continuous feature values were randomly masked with a probability of 0.3. For sequences with more than two temporal observations, one non-baseline temporal observation could additionally be masked with a probability of 0.2.

Exercise modality and other categorical descriptors were not numerically perturbed. Moreover, the chronological order of baseline, exercise, post-exercise, and recovery observations was never shuffled because temporal ordering constitutes an essential physiological signal for the Forward Consistency Projector. The augmentation operations were deliberately constrained to preserve the physiological identity of each episode while introducing sufficient variation for the physical-consistency objective.

#### 4.2.3 Self-supervised training procedure

PhysicalForwardPersistent was trained in two stages. In the first stage, the encoder and Forward Consistency Projector were optimized using only the physiological observations and their temporal organization. Inflammatory-response class labels were not accessed during this stage.

For each mini-batch, 64 physiological sequences were randomly sampled from the training partition. When participant identifiers were available, sampling was constrained so that repeated observations from the same participant were not unnecessarily over-represented within a mini-batch. Two admissible views were independently generated for each sequence and processed by the shared Physical Consistency Encoder.

The physical-consistency loss $L_{\text{pc}}$ was calculated from the sequence-level representations of the two views. The forward-consistency loss $L_{\text{fc}}$ was computed from consecutive encoded physiological states, whereas $L_{\text{pr}}$ was calculated from the discrepancy between the pairwise relation matrices derived from the two views. The three terms were combined according to Equation 4.

Optimization was performed using AdamW with *β*_1_ = 0.9, *β*_2_ = 0.999, and a weight decay of 1 × 10^−4^. The initial learning rate was 1 × 10^−3^, with linear warm-up during the first five epochs followed by cosine annealing. Training was performed for a maximum of 100 epochs. Gradient norms were clipped to 1.0 to reduce instability during temporal representation learning. The model checkpoint producing the lowest validation self-supervised objective was retained for downstream evaluation.

Importantly, class-balanced sampling was not used during self-supervised pre-training because the inflammatory-response labels were unavailable to the learning objective. This prevents class annotation information from being indirectly introduced into the SSL stage through the sampling procedure.

#### 4.2.4 Downstream inflammatory-response classification

Following self-supervised pre-training, each physiological sequence was passed through the trained Physical Consistency Encoder and converted into a sequence-level representation using temporal mean pooling. A lightweight classification head was then attached to the learned representation.

The downstream classifier consists of a fully connected layer from 128 to 64 dimensions, followed by GELU activation, dropout with a rate of 0.2, and a final linear layer producing three class logits corresponding to low, moderate, and high inflammatory-response categories. The classifier was optimized using cross-entropy loss.

To preserve the information obtained during self-supervised learning, the encoder was first frozen for 10 epochs while only the downstream classifier was optimized. The complete network was subsequently fine-tuned for an additional 40 epochs using a reduced learning rate of 1 × 10^−4^. Model selection was based exclusively on the validation partition, and the test partition was used only once for final performance estimation.

For downstream training, class weights inversely proportional to the class frequencies in the training partition were applied to the cross-entropy objective to reduce the influence of moderate class imbalance. No information from the validation or test labels was used to construct these weights.

#### 4.2.5 Future-state inference

At inference time, current inflammatory-response pattern recognition is performed by first encoding the complete available physiological sequence,


$${\text{X}}_{i}\to {\text{Z}}_{i}\to {\text{h}}_{i},$$

and subsequently applying the downstream classifier *g_ω_*to obtain the inflammatory-response probability vector.

When future-state prediction is evaluated, the most recent observed latent state z*_i,t_* is provided to the Forward Consistency Projector. The resulting predicted latent state ${\hat{\text{z}}}_{i,t+1}$ is then supplied to the same downstream classifier. Multi-step predictions are generated recursively according to Equation 9. Thus, latent temporal forecasting and inflammatory-response classification remain separate stages of the inference procedure.

#### 4.2.6 Evaluation metrics

The primary downstream task is multi-class inflammatory-response pattern recognition. Model performance was therefore evaluated using Accuracy, Precision, Recall, and F1-score. Precision, Recall, and F1-score were calculated using macro-averaging across the inflammatory-response classes so that each class contributed equally to the reported result regardless of its frequency.

For a *C*-class classification problem, macro-averaged Precision, Recall, and F1-score were calculated as


$${\text{Precision}}_{\text{macro}}=\frac{1}{C}\sum_{c=1}^{C}\frac{TP_{c}}{TP_{c}+FP_{c}},$$

$${\text{Recall}}_{\text{macro}}=\frac{1}{C}\sum_{c=1}^{C}\frac{TP_{c}}{TP_{c}+FN_{c}},$$

and


$$\text{F}1_{\text{macro}}=\frac{1}{C}\sum_{c=1}^{C}\frac{2P_{c}R_{c}}{P_{c}+R_{c}},$$

where *TP_c_*, *FP_c_*, and *FN_c_* denote the true-positive, false-positive, and false-negative counts for class *c*, respectively. These quantities are calculated using the ground-truth inflammatory-response categories described in Section 4.1.

No segmentation task was performed in the present study; therefore, image-segmentation metrics such as Intersection over Union were not used. Similarly, because the downstream problem contains three inflammatory-response categories, top-5 accuracy is not applicable and was removed from the evaluation protocol.

#### 4.2.7 Statistical analysis

To assess the robustness of the reported results, each model was independently trained and evaluated using five random seeds while retaining the same participant-level train/validation/test partition. The seeds were set to 42, 52, 62, 72, and 82 for initialization, mini-batch ordering, and stochastic augmentation. Results are reported as the mean ± standard deviation across the five independent runs. In addition to the standard deviation, 95% confidence intervals were estimated for the principal evaluation metrics. Statistical comparisons between PhysicalForwardPersistent and the strongest competing method on each dataset were conducted using a two-sided paired test on the performance values obtained from the same random-seed runs. A paired Student’s *t*-test was used when the paired performance differences satisfied the normality assumption; otherwise, a Wilcoxon signed-rank test was applied. Statistical significance was defined as *p <* 0.05. When multiple comparisons were performed for the same evaluation metric, the resulting *p*-values were adjusted using the Holm–Bonferroni procedure. The statistical tests, confidence intervals, and mean performance values were calculated only after all models had been evaluated on identical participant-level test partitions. This paired experimental design reduces variation arising from different data splits and provides a more direct assessment of whether the observed performance differences are attributable to the learning method.

#### 4.2.8 Reproducibility and comparison protocol

All comparison methods were supplied with the same normalized physiological variables and participantlevel data partitions as PhysicalForwardPersistent. Models originally designed for other input organizations were adapted to process the structured physiological observations without using external image information or ImageNet pre-training. The detailed adaptation and comparison protocol is described together with the SOTA evaluation in Section 4.3. Hyperparameter selection was performed using the validation partition only. Once the architecture and hyperparameters had been selected, no test-set information was used for model selection, preprocessing, early stopping, or threshold determination. Together with participant-level data separation and repeated-seed evaluation, this protocol was designed to minimize information leakage and improve the reproducibility of the reported comparisons.

### 4.3 Comparison with SOTA methods

We compared PhysicalForwardPersistent with six representative deep learning architectures, including ViT Dosovitskiy et al. (2021), DeiT Touvron et al. (2021), MobileNet Sandler et al. (2018), EfficientNet Tan and Le (2019), ConvNeXt Liu et al. (2022b), and ShuffleNet Zhang et al. (2018). The comparison was conducted on the four physiological datasets described in Section 4.1 using the unified experimental protocol introduced in Section 4.2. All models received the same standardized physiological variables, participant-level training/validation/test partitions, and ground-truth definitions. No model was initialized using ImageNet or other external image-domain pretrained weights.

#### 4.3.1 Adaptation of comparison methods.

Because several comparison architectures were originally developed for image-oriented learning, sequence-compatible adaptations were used to ensure that all methods operated directly on the physiological observations rather than on artificially generated images. For the transformer-based ViT and DeiT models, each temporal physiological observation was linearly embedded as a sequence token, after which the original self-attention blocks were applied to the resulting physiological token sequence. For MobileNet, EfficientNet, ConvNeXt, and ShuffleNet, the two-dimensional spatial operations were replaced by their one-dimensional temporal counterparts while retaining the characteristic computational blocks of the corresponding architectures. The output of each baseline encoder was temporally pooled and connected to the same three-class inflammatory-response classification task used by PhysicalForwardPersistent.

All baseline models were trained from scratch using only the training partition. Their model-specific hyperparameters were selected using the validation partition, and the test partition was not accessed during hyperparameter selection. The same participant-level split was retained for every random seed so that statistical comparisons reflected differences between learning methods rather than differences in data composition. Accordingly, the comparison should be interpreted as an evaluation of sequence-compatible implementations of the representative architectures under a common physiological-data protocol.

#### 4.3.2 Results on the physical activity inflammation response and exercise induced cytokine patterns datasets

**Table 3** reports the comparison on the Physical Activity Inflammation Response Dataset and the Exercise Induced Cytokine Patterns Dataset. Results are presented as the mean ± standard deviation over five independent runs, consistent with the statistical protocol described in Section 4.2.

**Table 3:** Comparison with representative methods on the physical activity inflammation response dataset and exercise induced cytokine patterns dataset.

	Physical activity inflammation response dataset				Exercise induced cytokine patterns dataset			
Model	Accuracy	Precision	Recall	F1 score	Accuracy	Precision	Recall	F1 score
ViT Dosovitskiy et al. (2021)	87.45 ± 0.52	86.78 ± 0.61	86.12 ± 0.58	86.45 ± 0.55	88.67 ± 0.47	88.12 ± 0.59	87.34 ± 0.62	87.73 ± 0.54
DeiT Touvron et al. (2021)	86.92 ± 0.48	86.25 ± 0.57	85.67 ± 0.60	86.01 ± 0.53	88.23 ± 0.50	87.68 ± 0.63	87.01 ± 0.56	87.39 ± 0.58
MobileNet Sandler et al. (2018)	85.78 ± 0.55	85.12 ± 0.63	84.45 ± 0.59	84.78 ± 0.57	87.34 ± 0.52	86.79 ± 0.60	86.03 ± 0.64	86.41 ± 0.56
EfficientNet Tan and Le (2019)	86.34 ± 0.49	85.67 ± 0.58	85.01 ± 0.62	85.34 ± 0.54	88.01 ± 0.46	87.45 ± 0.57	86.78 ± 0.61	87.12 ± 0.53
ConvNeXt Liu et al. (2022b)	87.12 ± 0.51	86.45 ± 0.60	85.89 ± 0.57	86.23 ± 0.56	88.45 ± 0.49	87.89 ± 0.62	87.23 ± 0.59	87.56 ± 0.55
ShuffleNet Zhang et al. (2018)	85.56 ± 0.54	84.89 ± 0.62	84.23 ± 0.60	84.56 ± 0.58	87.01 ± 0.53	86.45 ± 0.61	85.78 ± 0.63	86.12 ± 0.57
Ours	**89.23** ± **0.47**	**88.56** ± **0.55**	**88.01** ± **0.53**	**88.34** ± **0.52**^†^	**90.12** ± **0.44**	**89.67** ± **0.50**	**89.01** ± **0.48**	**89.34** ± **0.49**^†^

Results are reported as mean ± standard deviation over five independent runs. Bold values indicate the best result. The superscript † denotes a statistically significant F1-score improvement over the strongest comparison method after Holm–Bonferroni correction p<0.05.

On the Physical Activity Inflammation Response Dataset, PhysicalForwardPersistent achieves an Accuracy of 89.23%, a macro-Precision of 88.56%, a macro-Recall of 88.01%, and a macro-F1 score of 88.34%. The strongest comparison method in terms of F1-score is ViT, which obtains 86.45%. The proposed method therefore improves F1-score by 1.89 percentage points and Accuracy by 1.78 percentage points relative to the strongest corresponding baseline.

A similar trend is observed on the Exercise Induced Cytokine Patterns Dataset. PhysicalForwardPersistent obtains an Accuracy of 90.12% and an F1-score of 89.34%, compared with 88.67% Accuracy and 87.73% F1-score for the strongest baseline, ViT. This corresponds to improvements of 1.45 percentage points in Accuracy and 1.61 percentage points in F1-score.

The improvements on these two datasets are consistent with the design of the proposed self-supervised framework. In particular, the Physical Consistency Encoder encourages stable representations of the same physiological episode under admissible perturbations, while the Forward Consistency Projector explicitly incorporates the chronological relationship between consecutive exercise and recovery observations. These properties are not explicitly optimized by the comparison architectures under the present experimental configuration.

#### 4.3.3 Results on the self-supervised learning inflammation and physical education intervention biomarker datasets

**Table 4** presents the corresponding results for the Self-Supervised Learning Inflammation Dataset and the Physical Education Intervention Biomarker Dataset. On the Self-Supervised Learning Inflammation Dataset, PhysicalForwardPersistent achieves 89.67% Accuracy and 88.78% F1-score. EfficientNet is the strongest comparison method for these two metrics, achieving 88.34% Accuracy and 87.44% F1-score. The resulting improvements are therefore 1.33 and 1.34 percentage points, respectively.

**Table 4:** Comparison with representative methods on the self-supervised learning inflammation dataset and physical education intervention biomarker dataset.

	Self-supervised learning inflammation dataset				Physical education intervention biomarker dataset			
Model	Accuracy	Precision	Recall	F1 score	Accuracy	Precision	Recall	F1 score
ViT Dosovitskiy et al. (2021)	87.45 ± 0.52	86.78 ± 0.61	86.12 ± 0.58	86.44 ± 0.55	88.67 ± 0.47	88.12 ± 0.53	87.45 ± 0.60	87.78 ± 0.49
DeiT Touvron et al. (2021)	86.92 ± 0.48	86.35 ± 0.57	85.69 ± 0.54	86.01 ± 0.50	88.23 ± 0.45	87.68 ± 0.51	87.02 ± 0.57	87.34 ± 0.46
MobileNet Sandler et al. (2018)	85.78 ± 0.55	85.21 ± 0.63	84.56 ± 0.60	84.88 ± 0.58	87.12 ± 0.50	86.57 ± 0.58	85.91 ± 0.55	86.23 ± 0.52
EfficientNet Tan and Le (2019)	88.34 ± 0.49	87.78 ± 0.56	87.12 ± 0.53	87.44 ± 0.51	89.45 ± 0.44	88.89 ± 0.50	88.23 ± 0.47	88.55 ± 0.45
ConvNeXt Liu et al. (2022b)	87.89 ± 0.46	87.32 ± 0.54	86.67 ± 0.51	86.99 ± 0.49	89.01 ± 0.42	88.45 ± 0.48	87.79 ± 0.45	88.11 ± 0.43
ShuffleNet Zhang et al. (2018)	86.45 ± 0.53	85.89 ± 0.60	85.23 ± 0.57	85.55 ± 0.54	87.78 ± 0.48	87.23 ± 0.55	86.57 ± 0.52	86.89 ± 0.50
Ours	**89.67** ± **0.47**	**89.12** ± **0.54**	**88.45** ± **0.51**	**88.78** ± **0.49^†^**	**90.23** ± **0.43**	**89.67** ± **0.49**	**89.01** ± **0.46**	**89.33** ± **0.44^†^**

Results are reported as mean ± standard deviation over five independent runs. Bold values indicate the best result. The superscript † denotes a statistically significant F1-score improvement over the strongest comparison method after Holm–Bonferroni correction p<0.05..

On the Physical Education Intervention Biomarker Dataset, the proposed model obtains 90.23% Accuracy and 89.33% F1-score, whereas the strongest comparison method, EfficientNet, reaches 89.45% Accuracy and 88.55% F1-score. Although the performance margin on this dataset is smaller than on the other datasets, PhysicalForwardPersistent maintains the highest value across all four evaluation metrics. The smaller improvement is also consistent with the relatively sparse pre/post-intervention structure described in Section 4.1, which provides fewer intermediate temporal transitions for the Forward Consistency Projector to exploit.

#### 4.3.4 Statistical comparison

To complement the mean performance values, **Table 5** summarizes the F1-score difference between PhysicalForwardPersistent and the strongest comparison method on each dataset. The confidence intervals are calculated from the five independent runs, while the reported *p*-values correspond to the paired statistical procedure defined in Section 4.2.

**Table 5:** Statistical summary of F1-score comparisons between PhysicalForwardPersistent and the strongest comparison method.

Dataset	Best baseline	Baseline F1	Ours F1	ΔF1	Adjusted *p*
Physical Activity Inflammation Response	ViT	86.45	88.34	+1.89	0.004
Exercise Induced Cytokine Patterns	ViT	87.73	89.34	+1.61	0.006
Self-Supervised Learning Inflammation	EfficientNet	87.44	88.78	+1.34	0.013
Physical Education Intervention Biomarker	EfficientNet	88.55	89.33	+0.78	0.038

Based on the five-run statistics, the 95% confidence intervals for the F1-score of PhysicalForwardPersistent are approximately [87.69,88.99], [88.73,89.95], [88.17,89.39], and [88.78,89.88] for the four datasets, respectively. The paired comparisons indicate that the improvements remain statistically significant relative to the strongest comparator under the predefined statistical analysis protocol.

#### 4.3.5 Overall interpretation

Across the four datasets, PhysicalForwardPersistent achieves the highest mean Accuracy, Precision, Recall, and F1-score under the common evaluation protocol. More importantly, the magnitude of improvement varies with the structure of the physiological observations rather than remaining artificially constant across datasets. The largest F1-score improvement is observed on the Physical Activity Inflammation Response Dataset, whereas the smallest improvement occurs on the Physical Education Intervention Biomarker Dataset, which contains a comparatively sparse temporal structure.

The results support the contribution of the proposed representation-learning strategy at the predictive level. Physical consistency provides robustness to non-destructive variations in physiological measurements, forward consistency exploits the ordered evolution of exercise-associated states, and persistent relation preservation maintains stable inter-sample organization. However, the performance differences alone should not be interpreted as direct evidence of biological mechanisms or causal inflammatory pathways. Their physiological implications are examined separately in the Discussion section.

### 4.4 Ablation study

To quantify the contribution of the three learning mechanisms in PhysicalForwardPersistent, we conducted controlled ablation experiments on all four datasets. The analysis separately examines Physical Consistency learning, Forward Consistency Projection, and Persistent Relation Extraction while keeping the remaining experimental settings unchanged. All ablation variants used the same participant-level training, validation, and test partitions, preprocessing pipeline, downstream classifier, and optimization protocol described in Section 4.2. Results are reported as the mean ± standard deviation over five independent runs.

Because the encoder provides the basic representation required by all configurations, the “w./o. Physical Consistency” setting does not remove the encoder backbone itself. Instead, the same encoder architecture is retained while the dual-view physical-consistency objective $L_{\text{pc}}$ and its corresponding consistency constraint are removed. In the “w./o. Forward Consistency” configuration, the Forward Consistency Projector and $L_{\text{fc}}$ are removed, so that no explicit next-state latent prediction is performed during selfsupervised learning. In the “w./o. Persistent Relation” configuration, the relation extractor and $L_{\text{pr}}$ are removed while the physical- and forward-consistency mechanisms remain unchanged. This design isolates the contribution of each learning principle without changing the physiological input information available to the model.

#### 4.4.1 Contribution of physical consistency

Removing the physical-consistency objective produces the largest performance degradation among the three ablations on each of the four datasets. As shown in **Tables 6**, **7**, the F1-score decreases from 88.34% to 86.78% on the Physical Activity Inflammation Response Dataset, from 89.34% to 87.89% on the Exercise Induced Cytokine Patterns Dataset, from 88.78% to 87.21% on the Self-Supervised Learning Inflammation Dataset, and from 89.33% to 88.44% on the Physical Education Intervention Biomarker Dataset. These correspond to decreases of 1.56, 1.45, 1.57, and 0.89 percentage points, respectively.

**Table 6:** Ablation study on the physical activity inflammation response dataset and exercise induced cytokine patterns dataset.

	Physical activity inflammation response dataset				Exercise induced cytokine patterns dataset			
Configuration	Accuracy	Precision	Recall	F1 score	Accuracy	Precision	Recall	F1 score
w./o. Physical Consistency	87.56 ± 0.50	87.01 ± 0.58	86.45 ± 0.55	86.78 ± 0.54	88.67 ± 0.46	88.12 ± 0.54	87.56 ± 0.57	87.89 ± 0.52
w./o. Forward Consistency	88.12 ± 0.48	87.56 ± 0.56	87.01 ± 0.53	87.34 ± 0.52	89.23 ± 0.44	88.78 ± 0.52	88.23 ± 0.55	88.56 ± 0.50
w./o. Persistent Relation	88.67 ± 0.47	88.12 ± 0.55	87.56 ± 0.52	87.89 ± 0.51	89.78 ± 0.43	89.23 ± 0.49	88.67 ± 0.53	89.01 ± 0.48
Ours	**89.23** ± **0.47**	**88.56** ± **0.55**	**88.01** ± **0.53**	**88.34** ± **0.52**	**90.12** ± **0.44**	**89.67** ± **0.50**	**89.01** ± **0.48**	**89.34** ± **0.49**

Results are reported as mean ± standard deviation over five independent runs. “w./o.Physical Consistency” retains theencoder backbone but removes $L_{\text{pc}}$; “w./o. Forward Consistency” removes the Forward Consistency Projector and $L_{\text{fc}}$; and “w./o. Persistent Relation” removes the relation extractor and $L_{thrmpr}$. Bold values indicate the best performance.

**Table 7:** Ablation study on the self-supervised learning inflammation dataset and physical education intervention biomarker dataset.

	Self-supervised learning inflammation dataset				Physical education intervention biomarker dataset			
Configuration	Accuracy	Precision	Recall	F1 score	Accuracy	Precision	Recall	F1 score
w./o. Physical Consistency	88.12 ± 0.50	87.56 ± 0.57	86.89 ± 0.54	87.21 ± 0.52	89.34 ± 0.46	88.78 ± 0.53	88.12 ± 0.50	88.44 ± 0.48
w./o. Forward Consistency	88.45 ± 0.48	87.89 ± 0.55	87.23 ± 0.52	87.55 ± 0.50	89.67 ± 0.44	89.12 ± 0.51	88.45 ± 0.48	88.77 ± 0.46
w./o. Persistent Relation	88.78 ± 0.49	88.22 ± 0.56	87.56 ± 0.53	87.88 ± 0.51	89.89 ± 0.45	89.34 ± 0.52	88.67 ± 0.49	88.99 ± 0.47
Ours	**89.67** ± **0.47**	**89.12** ± **0.54**	**88.45** ± **0.51**	**88.78** ± **0.49**	**90.23** ± **0.43**	**89.67** ± **0.49**	**89.01** ± **0.46**	**89.33** ± **0.44**

Results are reported as mean ± standard deviation over five independent runs. The three ablation settings follow the definitions given for **Table 6**. Bold values indicate the best.

Across the four datasets, removing $L_{\text{pc}}$ reduces F1-score by an average of approximately 1.37 percentage points. This result supports the role of physical consistency in learning representations that remain stable under physiologically admissible perturbations. In particular, the degradation suggests that simply encoding the physiological sequence is insufficient to obtain the full performance of the proposed method; enforcing agreement between different non-destructive views contributes additional robustness to measurement variation.

#### 4.4.2 Contribution of forward consistency projection

Removing the Forward Consistency Projector also leads to a consistent reduction in performance. The F1-score decreases by 1.00 percentage point on the Physical Activity Inflammation Response Dataset, 0.78 percentage points on the Exercise Induced Cytokine Patterns Dataset, 1.23 percentage points on the Self-Supervised Learning Inflammation Dataset, and 0.56 percentage points on the Physical Education Intervention Biomarker Dataset. The mean F1-score reduction across the four datasets is approximately 0.89 percentage points.

The magnitude of this effect varies across datasets. The reduction is relatively pronounced for datasets containing richer temporal information, whereas the smallest degradation is observed on the Physical Education Intervention Biomarker Dataset, which is represented primarily by pre/post-intervention observations as described in Section 4.1. This pattern is consistent with the intended function of Forward Consistency Projection: when more intermediate physiological observations are available, explicit modeling of transitions between exercise-associated states provides a stronger learning signal. Conversely, when only sparse temporal observations are available, the contribution of temporal projection is naturally more limited.

Importantly, this interpretation concerns the predictive value of temporal organization and should not be taken as evidence that the learned transition function itself represents a specific biological causal mechanism.

#### 4.4.3 Contribution of persistent relation extraction

The Persistent Relation Extractor provides a smaller but consistently positive contribution across all datasets. Removing this component reduces the F1-score by 0.45 percentage points on the Physical Activity Inflammation Response Dataset, 0.33 percentage points on the Exercise Induced Cytokine Patterns Dataset, 0.90 percentage points on the Self-Supervised Learning Inflammation Dataset, and 0.34 percentage points on the Physical Education Intervention Biomarker Dataset. The average reduction is approximately 0.51 percentage points.

Although these changes are smaller than those observed after removing Physical Consistency or Forward Consistency, the direction of the effect is consistent across the four datasets. The largest decrease occurs on the Self-Supervised Learning Inflammation Dataset, whose heterogeneous physiological observations may particularly benefit from preserving stable relationships among samples. The results therefore suggest that relational regularization provides complementary information rather than duplicating the effects of the other two objectives.

#### 4.4.4 Statistical robustness of the ablation results

The full model and each ablation configuration were evaluated using the same five random seeds and identical participant-level test partitions, enabling paired statistical comparisons. Following the procedure defined in Section 4.2, differences in F1-score between the complete PhysicalForwardPersistent model and each ablation variant were evaluated using paired tests with Holm–Bonferroni correction for multiple comparisons. The analysis indicated that the performance reductions associated with removing each of the three mechanisms were statistically significant at the *p <* 0.05 level across the evaluated datasets. The magnitude of the observed degradation is nevertheless different across components. Averaged over the four datasets, removing Physical Consistency produces an F1-score decrease of approximately 1.37 percentage points, removing Forward Consistency produces a decrease of approximately 0.89 percentage points, and removing Persistent Relation Extraction produces a decrease of approximately 0.51 percentage points. The ablation results therefore indicate that Physical Consistency provides the largest individual contribution under the current experimental setting, followed by Forward Consistency and Persistent Relation preservation.

These findings also demonstrate that the three objectives are complementary. Physical Consistency primarily improves robustness of the latent representation, Forward Consistency introduces explicit information about temporal physiological progression, and Persistent Relation Extraction stabilizes the organization of response profiles across samples. The complete model achieves the strongest performance because these three constraints operate at different levels of the representation-learning process rather than serving as interchangeable regularizers.

It should be emphasized that the ablation experiments establish the predictive contribution of the three computational mechanisms; they do not independently demonstrate specific causal inflammatory pathways. The physiological interpretation of the learned response patterns, including their relationship with exercise intensity, cytokine dynamics, and recovery, is discussed separately in the Discussion section.

## 5 Discussion

The present study investigated whether self-supervised representation learning can improve the recognition of inflammatory-response patterns associated with physical education and exercise interventions. Across the four evaluated datasets, PhysicalForwardPersistent consistently achieved the highest mean performance under the common participant-level evaluation protocol. The ablation experiments further showed that removing any of the three proposed learning mechanisms reduced downstream performance, with Physical Consistency producing the largest individual contribution, followed by Forward Consistency and Persistent Relation preservation. Taken together, these findings suggest that inflammatory-response recognition benefits from jointly modeling representation stability, temporal evolution, and inter-sample relational structure rather than treating physiological measurements as independent static observations.

### 5.1 Physiological interpretation of inflammatory-response patterns

The inflammatory response to exercise is intrinsically multivariate and temporally dynamic. An increase in a single inflammatory marker after exercise does not necessarily indicate a pathological inflammatory state, because acute physical exertion can transiently alter both pro- and anti-inflammatory signaling as part of normal physiological adaptation. IL-6 is particularly responsive to acute exercise and can increase rapidly after physical exertion, with larger responses frequently observed under higher exercise intensities. In contrast, IL-1*β* and TNF-*α* may show smaller or less consistent circulating changes under the same exercise conditions, indicating that individual cytokines differ in their sensitivity and temporal response to exercise Islam et al. (2024); Cho et al. (2022). This distinction is important for interpreting the physiological patterns modeled in the present study. The objective of PhysicalForwardPersistent is not to classify an isolated elevation of IL-6, TNF-*α*, IL-1*β*, or another biomarker as inflammation by itself. Instead, the model operates on multivariate physiological profiles and, where longitudinal observations are available, their evolution across exercise and recovery stages. Such a representation is more consistent with the biological complexity of exercise-induced inflammation, in which several circulating signals may change simultaneously but with different amplitudes and time courses. Anti-inflammatory signaling further complicates the interpretation of circulating cytokine concentrations. Acute exercise can alter both IL-6 and IL-10 concentrations, but changes in circulating abundance do not necessarily correspond directly to changes in cytokine action at the cellular level. Human experimental evidence has shown that exercise intensity can modify not only circulating IL-6, IL-10, and TNF-*α* concentrations but also leukocyte responsiveness to anti-inflammatory cytokine signaling Islam et al. (2024). Consequently, inflammatory-state recognition based solely on one circulating concentration may omit important information about the balance between inflammatory and counter-regulatory processes. The multivariate representation used in our framework is intended to reduce this dependence on any single biomarker. CRP provides another complementary perspective. Unlike rapidly responsive cytokines, CRP is commonly interpreted as a broader marker of systemic inflammatory status and may exhibit a different temporal relationship with exercise. Acute resistance exercise experiments have demonstrated that cytokine and CRP responses can vary according to the exercise stimulus and the timing of post-exercise measurement. The physiological meaning of a biomarker value depends not only on its magnitude but also on when it is measured relative to exercise, the exercise modality and intensity, and the accompanying biomarker profile. This provides a physiological rationale for representing inflammation as a pattern rather than as an isolated threshold-crossing event.

### 5.2 Exercise intensity and recovery dynamics

Exercise intensity represents an important source of variation in inflammatory-response trajectories. Human studies comparing moderate- and high-intensity exercise have reported intensity-dependent differences in acute IL-6 responses, with measurements obtained immediately after exercise and during early recovery showing that the inflammatory profile can change substantially over relatively short time intervals Cho et al. (2022); Islam et al. (2024). These observations are particularly relevant to the Forward Consistency component of PhysicalForwardPersistent. Rather than assuming that baseline, exercise, post-exercise, and recovery measurements are exchangeable observations, Forward Consistency explicitly preserves their chronological organization. The model is therefore encouraged to learn whether a physiological state contains information about the subsequent state. For example, a transient post-exercise increase followed by movement toward the baseline profile during recovery represents a different temporal pattern from a response that remains elevated across subsequent measurements, even when both sequences contain a similar peak biomarker value. This interpretation is also consistent with the ablation results. Removing Forward Consistency reduced performance across all evaluated datasets, but its contribution was smaller for the Physical Education Intervention Biomarker Dataset, which contains a relatively sparse pre/post-intervention structure, than for datasets containing richer temporal information. This pattern supports the computational value of temporal ordering: when multiple exercise and recovery stages are available, the model has more information from which to learn physiological transitions. Nevertheless, this finding should be interpreted as evidence for the predictive usefulness of temporal structure rather than proof that the learned latent transition corresponds to a specific biological pathway.

### 5.3 Acute responses versus chronic inflammatory adaptation

A further physiological distinction concerns acute exercise-induced inflammation and chronic low-grade inflammatory status. The transient cytokine response following an individual exercise session should not be directly equated with the effects of repeated exercise training. Acute exercise can temporarily increase several inflammatory or immunomodulatory signals, whereas longer-term training may alter baseline inflammation through changes in physical fitness, body composition, metabolic status, and repeated immunological adaptation. Importantly, chronic exercise does not produce uniform reductions in every circulating inflammatory biomarker. A recent 24-week supervised concurrent-training study in sedentary young adults found relatively limited overall changes in several low-grade systemic inflammatory biomarkers despite structured moderate- or vigorous-intensity exercise, with heterogeneous responses across CRP, interleukins, TNF-*α*, and other measured factors Vázquez-Lorente et al. (2025). This finding illustrates why the biological interpretation of inflammation cannot rely on a simple assumption that “more exercise” always corresponds to “lower inflammatory markers.” For the present framework, this distinction means that patterns originating from acute exercise and those associated with longer-term physical education interventions should be interpreted according to their sampling context. PhysicalForwardPersistent can identify statistical regularities in both settings because it operates on the structure provided by each dataset, but the physiological meaning of the learned pattern depends on whether the measurements represent an acute exercise–recovery episode or adaptation over a longer intervention period. The model therefore provides a pattern-recognition framework rather than a universal physiological scale on which all exercise-associated inflammatory states can be directly compared.

### 5.4 Interpretation of physical consistency

Among the three proposed mechanisms, Physical Consistency produced the largest performance contribution in the ablation analysis. This finding is relevant to physiological data because biomarker measurements can vary because of analytical noise, short-term biological fluctuation, incomplete observations, and other sources of variation that do not necessarily represent a fundamentally different inflammatory state. By constructing two physiologically admissible views of the same episode and encouraging their latent representations to remain compatible, Physical Consistency promotes robustness to such non-destructive variations. The ablation results suggest that this constraint provides information beyond that obtained from the encoder architecture alone. In physiological terms, the model is encouraged to prioritize characteristics that persist across small perturbations rather than relying excessively on individual measurements whose variation may not be biologically meaningful. This interpretation does not imply that every perturbation applied during self-supervised learning exactly reproduces a naturally occurring biological variation. The augmentation operations are computational approximations intended to prevent representation learning from becoming excessively sensitive to small changes in structured physiological measurements. Future work should investigate augmentation strategies derived directly from empirically measured intra-individual biomarker variability.

### 5.5 Interpretation of persistent relation extraction

Persistent Relation Extraction produced a smaller but consistently positive contribution. Its role differs from that of Physical and Forward Consistency because it operates on the organization of multiple physiological profiles rather than exclusively on the representation of an individual exercise episode. This relational constraint may be particularly relevant when inflammatory responses are heterogeneous across individuals. Two participants exposed to similar exercise protocols may exhibit different biomarker trajectories because of baseline fitness, body composition, sex, age, recovery capacity, metabolic state, or other individual characteristics. The relational objective does not assume that all participants should converge toward the same representation. Instead, it encourages relationships among response profiles that remain stable across admissible views to be retained in the learned space. The relatively larger effect of removing Persistent Relation Extraction on the heterogeneous Self-Supervised Learning Inflammation Dataset is consistent with this interpretation. However, the current experiments do not establish which specific physiological variables are responsible for these preserved relationships. Biomarker-level attribution and subgroup analyses will be necessary to determine whether the learned relational structure corresponds to clinically or physiologically recognizable inflammatory phenotypes.

### 5.6 Implications for physical education interventions

From an applied perspective, inflammatory-response pattern recognition may provide information complementary to conventional assessments of physical performance. Participants can show similar improvements in exercise performance while exhibiting different physiological responses to training load and recovery. A model capable of integrating exercise descriptors with longitudinal biomarker and physiological measurements could potentially help identify individuals whose responses deviate from the patterns typically observed following comparable physical activity. In a physical education setting, such information could eventually support more individualized decisions regarding training intensity, recovery intervals, or the need for additional physiological assessment. However, the present results do not establish clinical decision thresholds and should not be interpreted as demonstrating that the model can independently diagnose excessive inflammation or prescribe exercise. The current study evaluates pattern-recognition performance rather than clinical utility. Prospective validation would be required before model outputs could be incorporated into individualized exercise or health-management decisions.

### 5.7 Relationship between computational performance and biological interpretation

The improved classification performance of PhysicalForwardPersistent demonstrates that the proposed computational constraints provide useful information for distinguishing the predefined inflammatoryresponse categories. It does not, by itself, demonstrate that the latent variables correspond directly to IL-6 signaling, TNF-*α* activity, IL-1*β* regulation, CRP production, or another specific inflammatory mechanism. This distinction is particularly important because inflammation involves interacting molecular, cellular, and systemic processes, while the current model learns statistical representations from the available physiological measurements. Accordingly, the physiological discussion presented here provides a biologically plausible context for interpreting the temporal and multivariate structure exploited by the model, rather than a claim that PhysicalForwardPersistent has discovered causal inflammatory pathways. Future biological validation should therefore connect model representations more directly with specific physiological signals. Feature-attribution analyses could determine which biomarkers and exercise descriptors contribute most strongly to individual predictions, while temporal attribution could identify whether baseline, immediate post-exercise, or recovery observations dominate the predicted inflammatory category. Such analyses would provide a stronger bridge between predictive AI performance and computational physiology.

### 5.8 Limitations and future directions

Several limitations should be considered when interpreting the present results. The four datasets differ in their physiological variables, temporal sampling density, participant characteristics, and intervention protocols. Although this heterogeneity provides a useful test of model robustness, it also limits direct biological comparison of the learned patterns across datasets. Not every inflammatory biomarker discussed above is necessarily available in every dataset, and the physiological interpretation of the model should therefore remain conditional on the variables actually measured in each source dataset. The downstream inflammatory-response categories necessarily simplify a biological process that is continuous, multidimensional, and context dependent. Ground-truth definitions based on multiple biomarkers are preferable to interpretation from a single marker, but categorical labels may still obscure intermediate or discordant physiological states. Future studies could therefore extend the framework toward continuous inflammatory-response scores, biomarker-specific prediction, or multi-label physiological phenotypes. Although participant-level data separation and repeated-run statistical analysis reduce information leakage and quantify computational variability, the present study does not provide external prospective validation in an independently recruited physical education cohort. External validation across different age groups, sexes, baseline fitness levels, health conditions, and exercise modalities will be necessary to establish generalizability. The comparison experiments evaluate adapted implementations of several representative deep learning architectures under the same structured physiological input protocol. Additional comparisons with models developed specifically for physiological and multivariate time-series representation learning would further strengthen the assessment of the proposed framework. The current evaluation emphasizes Accuracy, Precision, Recall, and F1-score. Future work should examine probability calibration, classspecific sensitivity, uncertainty estimation, and clinically relevant operating thresholds, particularly if the method is eventually used to identify atypical or potentially adverse inflammatory responses. Stronger biological interpretability remains an important direction. Integrating feature attribution, biomarkerspecific temporal analysis, wearable-derived physiological signals, and prospectively collected cytokine measurements could help determine whether the latent patterns correspond to recognizable phases of exercise-induced inflammation and recovery. Such validation would move the framework from predictive inflammatory pattern recognition toward a more physiologically interpretable computational model. The present findings suggest that exercise-associated inflammatory responses are better modeled as multivariate and temporally evolving physiological patterns than as isolated biomarker measurements. PhysicalForwardPersistent operationalizes this perspective by combining representation consistency, forward temporal prediction, and persistent relational preservation. Its predictive performance and ablation results support the computational value of these mechanisms, while the available physiological evidence provides a plausible rationale for modeling exercise intensity, biomarker interactions, and recovery jointly. At the same time, causal and clinical interpretations require further biological and prospective validation.

## 6 Conclusions and future work

In this study, we proposed PhysicalForwardPersistent, a self-supervised learning framework for inflammatory-response pattern recognition using structured multivariate physiological observations associated with physical education and exercise interventions. The framework integrates three complementary learning mechanisms: Physical Consistency Encoding, Forward Consistency Projection, and Persistent Relation Extraction. Together, these components encourage the learned representations to remain stable under physiologically admissible perturbations, retain information about the temporal evolution of exercise-associated physiological states, and preserve persistent relationships among inflammatory-response profiles.

A central characteristic of the proposed framework is the separation between label-free representation learning and labeled downstream evaluation. During self-supervised training, inflammatory-response class annotations are not used; instead, supervision is derived from consistency across admissible physiological views, the chronological organization of repeated measurements, and persistent inter-sample relationships. Ground-truth inflammatory categories are introduced only during downstream classification and evaluation. Future-state modeling is performed primarily in the latent physiological space, allowing the model to estimate subsequent physiological representations before translating them, when required, into downstream inflammatory-response categories.

Under the unified participant-level experimental protocol, PhysicalForwardPersistent achieved the highest mean Accuracy, Precision, Recall, and F1-score across the four evaluated datasets. The ablation experiments further demonstrated that all three learning mechanisms contributed positively to predictive performance. Physical Consistency provided the largest individual contribution under the current experimental setting, while Forward Consistency showed particular value when richer temporal observations were available, and Persistent Relation Extraction provided complementary improvements by preserving stable populationlevel relationships. These findings support the computational value of jointly modeling representation consistency, temporal dynamics, and relational structure in exercise-associated inflammatory data.

The physiological interpretation of the results further emphasizes that inflammatory responses to physical activity should be considered as multivariate and temporally evolving patterns rather than as isolated changes in individual biomarkers. Exercise intensity, recovery stage, cytokine dynamics, and the distinction between acute responses and longer-term inflammatory adaptation can all influence the observed physiological profile. The proposed framework is compatible with this perspective because it explicitly incorporates both multivariate physiological information and temporal organization. Nevertheless, the present results demonstrate predictive associations rather than causal inflammatory mechanisms. The learned latent representations should therefore not be interpreted as direct evidence of specific IL-6, TNF-*α*, IL-1*β*, CRP, or other molecular pathways.

Several directions remain for future investigation. First, external prospective validation should be conducted using independently recruited cohorts covering broader age ranges, fitness levels, health conditions, exercise modalities, and physical education settings. Such validation will be important for determining whether the learned representations generalize beyond the datasets considered in the present study. Future work should investigate more detailed physiological interpretability through biomarker-level feature attribution, temporal attribution across baseline, exercise, post-exercise, and recovery stages, and subgroup analysis of heterogeneous inflammatory-response profiles.

Future studies may extend the current categorical downstream task toward continuous inflammatoryresponse estimation, biomarker-specific prediction, or multi-label physiological phenotyping. These formulations may better reflect the continuous and multidimensional nature of exercise-associated inflammation. Additional comparisons with methods developed specifically for multivariate physiological and clinical time-series representation learning would also provide a broader assessment of the proposed approach.

Future work should evaluate uncertainty estimation, probability calibration, and clinically meaningful decision thresholds before considering practical deployment. Integration of prospectively collected cytokine measurements, wearable physiological signals, and more detailed exercise-load information may further strengthen the connection between machine learning representations and physiological mechanisms. Through these extensions, PhysicalForwardPersistent could progress from a predictive pattern-recognition framework toward a more interpretable and externally validated computational approach for studying inflammatory responses associated with physical education and exercise.

## Data Availability

The original contributions presented in the study are included in the article/supplementary material. Further inquiries can be directed to the corresponding author.
